# Assessing the potential for biogeochemical deterioration of building materials by green algae in temperate climate via an integrated metabolomic approach

**DOI:** 10.1038/s41598-026-58452-8

**Published:** 2026-06-19

**Authors:** Paulina Nowicka-Krawczyk, Michał Komar, Beata Gutarowska, Tomasz Ruman, Joanna Nizioł, Joanna Żelazna-Wieczorek

**Affiliations:** 1 Department of Algology and Mycology, Faculty of Biology and Environmental Protection, University of Lodz, Poland, Banacha 12/16, 90-237 Lodz, Poland; 2 Department of Environmental Biotechnology, Faculty of Biotechnology and Food Sciences, Lodz University of Technology, Wólczańska 171/173, 90-530 Lodz, Poland; 3 Interdisciplinary Doctoral School, Lodz University of Technology, Żeromskiego 116, 90-924 Lodz, Poland; 4 Faculty of Chemistry, Rzeszów University of Technology, al. Powstańców Warszawy 6, 35-959 Rzeszów, Poland

**Keywords:** Aerial algae, Geochemical biodeterioration, Building materials, Metabolites, Biodeteriorative acids, Biochemistry, Biogeochemistry, Environmental sciences, Microbiology

## Abstract

Biodeterioration of building materials by aerophytic green algae is increasingly recognized as a significant factor affecting the aesthetic value and durability of brick and plaster façades, yet the biochemical mechanisms underlying this process remain poorly understood. This study investigates the metabolomic profiles of five algal taxa: *Chloroidium saccharophilum* PNK010, *Klebsormidium nitens* PNK013, *Bracteacoccus minor* PNK015, *Diplosphaera chodatii* PNK021, and *Stichococcus bacillaris* PNK040 growing on brick and plaster for a year under laboratory and environmental conditions. Untargeted UHPLC–MS/MS metabolomics, with metabolic pathway and chemical-class enrichment analyses, were used to characterize substrate- and condition-associated metabolic signatures and to detect metabolites potentially involved in material deterioration. The research demonstrated strain-specific and substrate-driven metabolic differentiation. Plaster exhibited higher metabolic heterogeneity and stress-related pathways, whereas brick showed more conserved, growth-associated metabolic profiles. Across taxa, low-molecular-weight organic acids, including citric/isocitric, acetic, and oxalic, were recurrently detected, suggesting their potential involvement in acidification- and metal-complexation-related processes at the algal–mineral interface. Enrichment of flavin nucleotides, carboxylic acids, fatty acyls, and aromatic secondary metabolites further supports the involvement of redox activity, stress adaptation, and biofilm persistence. These findings allow a provisional ranking of the algae based on their potential for geochemical biodeterioration, as follows: PNK010 > PNK040> PNK015 > PNK021> PNK013.

**Supplementary Information:**

The online version contains supplementary material available at https://doi.org/10.1038/s41598-026-58452-8.

## Introduction

Biodeterioration is generally understood as the undesirable alteration of materials resulting from the metabolic activity of living organisms. This concept, formally introduced in the mid-twentieth century, reflects a long-standing interaction between construction materials and the natural environment across diverse climatic and geographical contexts^1^. Among the various biotic agents involved, microorganisms are particularly challenging due to their ubiquity, metabolic diversity, and the difficulty of controlling their growth on exposed surfaces. Research on material biodeterioration has traditionally focused on heterotrophic microorganisms, particularly bacteria and fungi, whose capacity to decompose organic and inorganic substrates is well documented^2^. In the temperate climate zone, aerophytic (terrestrial or airborne) green algae are pioneer organisms that colonize exposed surfaces and can dominate terrestrial biofilms^3,4^. This group of algae is well adapted to life in harsh terrestrial environments, and their physiological dependence on liquid water is limited, as they can grow using only atmospheric moisture and nutrients deposited by aerosols and dust^5^. Representatives of green algae are known to inhabit terrestrial substrates, including bricks, plaster, concrete, stone, wood, and synthetic materials^3,4^. Following successful attachment, rapid cell division leads to the development of photosynthetic biofilms, with visible surface discoloration often appearing within several weeks and mature biofilm formation occurring after approximately 40–60 days^6^.

The development and persistence of phototrophic biofilms are governed by environmental factors, including light availability, temperature, relative humidity, nutrient availability, and substrate pH, which collectively shape seasonal dynamics on building surfaces^7,8^. While substrate type is not always considered a primary determinant of colonization, physical properties such as porosity and surface roughness strongly influence algal adhesion, water retention, and subsequent growth^9,10^.

Despite extensive research on the biodeterioration of natural stone, comparatively little attention has been paid to ceramic materials, such as brick and plaster, which differ significantly in mineral composition, pore structure, and durability. Given the long history of brick as a construction material and the widespread use of plastered façades in both historical and modern architecture, a deeper understanding of the interactions between terrestrial algae and these materials is essential.

Previous laboratory studies have often reported limited short-term effects of algal growth on bricks^11^. In contrast, more recent experiments indicate changes in surface pH, water absorption, and discoloration following algal colonization^12^. However, this research was conducted as a short-term experiment and confirmed that green phototrophic biofilm, with a mixed algal composition, adversely affects the structure of mineral substrates. Long-term studies confirming the biodeteriorative potential of particular taxa are very scarce.

The contribution of particular green algal strains, classified as widespread first colonizers of plaster and brick, i.e., *Chloroidium saccharophilum* PNK010, *Klebsormidium nitens* PNK013, *Bracteacoccus minor* PNK015, *Diplosphaera chodatii* PNK021, and *Stichococcus bacillaris* PNK040, to geophysical biodeterioration has been studied by Nowicka-Krawczyk et al.^13^. After six months of growth, all algal taxa except *B. minor* were able to detach the mineral parts of the brick surface as a result of the biofilm’s natural separation from the substrate.

To better understand these algae’s biodeteriorative mechanisms, a deeper investigation of their biochemical interactions with mineral substrates is essential. This will not only address the knowledge gap regarding how pioneer algae may affect the substrates they colonize, but also enable the assessment of algal-based biodeterioration risk, thereby informing the development of effective strategies for the protection and conservation of built heritage.

Based on previous studies and the literature, the hypothesis that widespread green algae may contribute to geochemical biodeterioration is justified. Therefore, this study focused on determining the metabolomic profiles of specific aerial algal taxa to identify potential chemical markers associated with plaster and brick deterioration. For the purpose of this research, unialgal cultures of algae previously studied for geophysical deteriorative potential^13^ were inoculated onto experimental plaster and brick, incubated under both laboratory and environmental conditions for one year, and then the metabolomic analyses were carried out on each taxon in relation to the substrate and cultivation conditions. Metabolomic pathway analysis allowed checking whether particular algae exhibit a conserved metabolic profile when growing on different substrates and under different conditions, while the enrichment analysis of substrates indicated the main chemical classes of metabolites that potentially affect the substrates as a result of biofilm growth.

To ensure a transparent and reproducible comparison of the biodeteriorative potential among the studied algae, a composite risk assessment framework was applied. This approach integrates (1) metabolite enrichment analysis, reflecting the overrepresented chemical classes potentially hazardous to substrates (e.g., organic acids, phenolics, lipids); (2) metabolic pathway analysis, indicating metabolic versatility; and (3) confirmed production of highly biodeteriorative organic acids (acetic, oxalic, and citric acids), representing direct drivers of mineral dissolution. In the final ranking, enrichment analysis was assigned the highest weight, followed by acid production and then pathway analysis.

## Results

### Aerial algal metabolomic profile analysis

As a result of the research, a total of 817 metabolites were annotated in the investigated strain samples: 292 in Negative Ion Mode and 525 in Positive Ion Mode (Supplementary Table S1). On plaster substrates (P), the differences in the number of metabolites between the laboratory (L) and environmental (E) culturing were strain-dependent, although for *C. saccharophilum* PNK010 and *S. bacillaris* PNK040, the predominance of metabolites in the environmental samples was evident (PNK010_L/E: 515/676; PNK040_L/E: 411/629). In contrast, on the brick (B), cultivation under laboratory conditions yielded higher metabolite counts than the growth under environmental conditions across all strains (PNK010_L/E: 607/553; PNK013_L/E: 603/502; PNK015_L/E: 613/560; PNK021_L/E: 577/527; PNK040_L/E: 586/493). Comparing the type of substrates, only the cultivation in the laboratory showed a clear trend that brick consistently supports a higher number of metabolites, regardless of strain (PNK010_P/B: 515/607; PNK013_P/B: 548/603; PNK015_P/B: 527/613; PNK021_P/B: 556/577; PNK040_P/B: 411/586) (Supplementary Tables S2–S21).

**Fig. 1:**
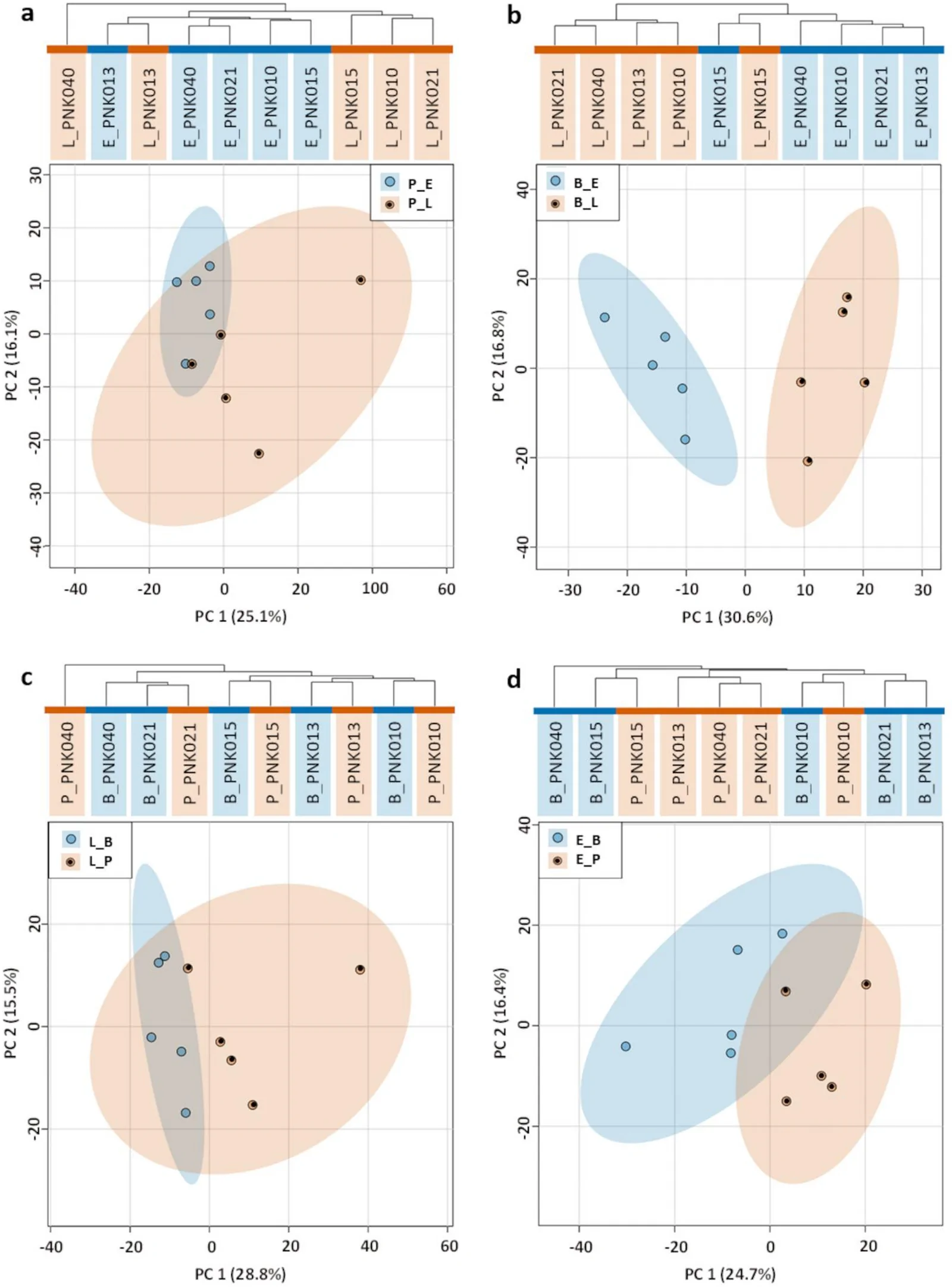
Principal component analysis (PCA) score plots (with ellipses as the 95% coincidence intervals) of metabolomic profiles for algal strains growing for a year on plaster (P) and brick (B) in laboratory (L) and environment (E) supported with hierarchical clustering of strains generated with heatmaps strains` metabolomic profiles (see: Supplementary Figs S1–S2); (**a**) profiles for algae growing on plaster (P) in relation to the site – laboratory (L) vs. environment (E); (**b**) profiles for algae growing on brick (B) in relation to the site – laboratory (L) vs. environment (E); (**c**) profiles for algae growing in laboratory (L) in relation to substrate type: plaster (P) vs. brick (B); (**d**) profiles for algae growing in environment (E) in relation to substrate type: plaster (P) vs. brick (B); strains refer to algal taxa: (PNK010) *Chloroidium saccharophilum*, (PNK013) *Klebsormidium nitens*, (PNK015) *Bracteacoccus minor*, (PNK021) *Diplosphaera chodatii*, (PNK040) *Stichococcus bacillaris*.

The Principal Component Analysis (PCA) of metabolomic profiles (Fig. 1) and hierarchical clustering of normalized metabolite intensities (Supplementary Figs S1–S2) enabled a more thorough investigation of the heterogeneity of metabolic profiles across samples. The analyses revealed pronounced differences among the five algal strains in terms of growth site and substrate type – metabolomic patterns were associated with both factors, with strain-specific responses evident across all comparisons.

On plaster, metabolic profiles of strains clearly differentiated between *ex situ* and in situ growth (Fig. 1a). For most strains, laboratory- and environment-grown samples formed separate clusters, suggesting a strong association between growth site and metabolite composition. This separation was particularly evident for *C. saccharophilum* PNK010, *B. minor* PNK015, and *D. chodatii* PNK021 strains, which displayed marked shifts in metabolite abundance between conditions. In contrast, *K. nitens* PNK013 exhibited a more conserved metabolic profile, with laboratory and environmental samples clustering more closely. The *S. bacillaris* PNK040 formed a distinct cluster under laboratory conditions, characterized by pronounced differences in metabolite intensities compared to its environmental counterpart.

On the brick, the influence of the growth site was also evident (Fig. 1:b). Samples clustered according to the growth environment, suggesting a recurrent environment-associated metabolomic pattern across strains. Nevertheless, strain-specific responses were retained, with *B. minor* PNK015 showing a clear separation between laboratory and environmental profiles. Overall, brick-grown samples exhibited lower contrast in metabolite abundance patterns than plaster ones (Supplementary Fig. S1), suggesting more constrained metabolic variability.

Under laboratory conditions, metabolic profiles clustered primarily by substrate type, with most strains forming distinct plaster- and brick-associated subclusters (Fig. 1c). This substrate-driven separation and metabolic fingerprint was highly pronounced for all algal strains (Supplementary Fig. S2). In environmental conditions, substrate-related differentiation remained evident but varied among strains (Fig. 1d). The *K. nitens* PNK013 and *D. chodatii* PNK021 clustered primarily by substrate, while *C. saccharophilum* PNK010, *B. minor* PNK015, and *S. bacillaris* PNK040 exhibited strong metabolic divergence between plaster and brick. Overall, plaster-grown environmental samples displayed greater heterogeneity in metabolite abundance than those grown on brick.

Among the detected metabolites, some organic acids known to have a biodeteriorative impact were identified. In the majority of samples, the citric acid and/or its stereoisomer D-threo-isocitric acid were detected. Only in the *B. minor* PNK015 and *S. bacillaris* PNK040, both from plaster in laboratory and environmental conditions, this compound was not detected. In three strains growing under laboratory conditions – *C. saccharophilum* PNK010 on plaster and brick, *K. nitens* PNK013, and *B. minor* PNK015, both growing on brick – the presence of acetic acid was detected. Moreover, oxalic acid, an organic acid with well-documented biodeteriorative relevance, was detected in profiles of four strains: *C. saccharophilum* PNK010 and *D. chodatii* PNK021, both growing on plaster but in different conditions (in situ and *ex situ*, respectively), and in *K. nitens* PNK013 and *B. minor* PNK015, both growing on brick in laboratory conditions.

### Screening the metabolic pathways of algae

After metabolite annotation using the KEGG *Chlorella variabilis* reference pathway database^14^, 63 metabolic pathways were mapped across all samples (Supplementary Table S22); however, 19 showed significant overrepresentation (*p* ≤ 0.05) in at least one sample (Table 1). The list of all annotated metabolic pathways in strain samples is presented in Supplementary Tables S23–S42, while the topology of pathways, with a focus on the most substantial, is visualized in Supplementary Figs S3–S7.

**Table 1:** Impact scores of metabolic pathways significantly overrepresented among annotated metabolites (*p* ≤ 0.05) in algal strains growing for a year on plaster and brick in laboratory and environmental conditions; pathway analysis for metabolites with fold change > 2 compared to the corresponding control samples was annotated using the KEGG *Chlorella variabilis* reference database^14^; numbers (0–1) are the impact values; dots (▪) exhibit non-significant impact (*p* > 0.05); strains refer to algal taxa: (PNK010) *Chloroidium saccharophilum*, (PNK013) *Klebsormidium nitens*, (PNK015) *Bracteacoccus minor*, (PNK021) *Diplosphaera chodatii*, (PNK040) *Stichococcus bacillaris*.

Pathway/metabolism	Alanine, aspartate, and glutamate	Arginine and proline	Arginine biosynthesis	Citrate cycle (TCA cycle)	Cysteine and methionine	Galactose	Glutathione	Glycerophospholipid	Glycine, serine, and threonine	Glyoxylate and dicarboxylate	Isoquinoline alkaloid biosynthesis	Nicotinate and nicotinamide	Nitrogen	One carbon pool by folate	Purine	Pyrimidine	Riboflavin	Starch and sucrose	Tyrosine
Strain																			
Plaster laboratory																			
PNK010	0.84	▪	0.52	▪	0.24	▪	0.57	0.31	0.45	0.37	1.0	▪	▪	0.18	0.44	▪	▪	▪	0.45
PNK013	0.84	▪	0.52	▪	0.24	▪	0.23	0.32	0.45	0.37	1.0	▪	▪	0.18	0.42	▪	▪	▪	0.45
PNK015	0.84	▪	0.52	▪	0.24	▪	0.54	0.32	0.45	0.28	1.0	▪	▪	▪	0.42	▪	▪	▪	0.45
PNK021	0.75	▪	0.52	▪	0.24	▪	0.58	0.32	0.45	0.34	1.0	0.12	▪	0.18	0.42	0.31	▪	▪	0.45
PNK040	▪	▪	0.38	▪	▪	▪	▪	0.29	▪	0.15	0.50	▪	▪	▪	0.26	▪	0.23	▪	▪
Plaster environment																			
PNK010	0.84	0.22	0.52	▪	▪	▪	▪	0.36	▪	0.30	1.0	0.12	▪	0.42	0.48	▪	▪	▪	0.45
PNK013	0.84	▪	0.52	▪	0.21	0.29	0.52	0.31	0.45	0.37	0.50	▪	0.27	0.12	▪	▪	▪	▪	0.42
PNK015	0.84	▪	0.52	▪	▪	0.29	0.52	0.34	0.45	0.37	0.50	▪	▪	▪	▪	▪	▪	▪	0.42
PNK021	0.84	▪	0.52	▪	▪	0.28	0.58	0.28	0.45	0.37	1.0	0.12	▪	0.18	0.40	▪	▪	▪	0.45
PNK040	0.84	0.22	0.52	▪	0.24	▪	0.58	0.25	0.55	0.28	1.0	0.12	▪	0.48	0.45	▪	▪	▪	▪
Brick laboratory																			
PNK010	0.84	▪	0.52	▪	0.24	▪	0.61	▪	0.55	0.37	0.50	0.09	▪	0.48	0.41	▪	▪	▪	0.42
PNK013	0.84	▪	0.52	▪	▪	0.16	0.58	0.32	0.45	0.37	1.0	▪	▪	0.12	0.44	▪	▪	▪	0.45
PNK015	0.84	▪	0.52	▪	▪	0.29	0.50	0.32	0.45	0.30	1.0	▪	▪	0.42	0.42	▪	▪	▪	0.45
PNK021	0.84	▪	0.52	▪	0.24	0.28	0.58	0.23	0.45	0.37	1.0	0.12	▪	0.18	0.45	▪	▪	▪	0.45
PNK040	0.84	▪	0.52	▪	0.24	▪	0.58	▪	0.45	0.37	1.0	0.09	▪	0.18	0.44	▪	▪	▪	0.45
Brick environment																			
PNK010	0.84	▪	0.52	▪	0.24	0.29	▪	0.31	▪	0.30	1.0	▪	▪	▪	0.40	0.27	▪	▪	0.45
PNK013	0.84	▪	0.52	0.18	0.21	0.29	▪	0.32	0.45	0.37	0.50	0.09	▪	0.13	0.44	0.36	▪	0.40	0.42
PNK015	0.84	▪	0.52	▪	0.21	▪	0.52	0.32	0.55	0.28	0.50	▪	▪	0.43	0.42	0.36	▪	▪	0.42
PNK021	0.84	▪	0.43	▪	0.22	0.29	▪	0.32	▪	0.37	1.0	0.12	▪	0.18	0.44	▪	▪	▪	0.45
PNK040	0.84	▪	0.38	▪	▪	0.29	▪	0.23	0.38	0.28	1.0	0.04	▪	0.13	0.30	▪	▪	0.40	0.45

Five metabolic pathways appeared repeatedly across algal strains, substrate types, and growing conditions and were overrepresented (*p* ≤ 0.05) in > 95% of samples (Table 1). The isoquinoline alkaloid biosynthesis pathway, indicated as substantial across all samples, had the highest overall impact, with many entries at 1.0 and some at 0.5. This pathway occurred without any discernible pattern across strains, substrates, or growth sites. The glyoxylate and dicarboxylate metabolism occurred with equal frequency, but its impact was lower, ranging from 0.28 to 0.37, with the lowest value observed in *S. bacillaris* PNK040 in the laboratory. The arginine biosynthesis pathway was also ubiquitous, with a moderate impact (mainly 0.5), but in *S. bacillaris* PNK040 in the laboratory, the impact was lower (0.38). Two other pathways were significant (*p* ≤ 0.05) across all strain samples, except for the aforementioned *S. bacillaris* PNK040, which was cultivated in the laboratory. The alanine, aspartate, and glutamate pathway had a stable impact (0.84) across all samples; however, in *D. chodatii* PNK021, it decreased slightly to 0.75. Tyrosine metabolism occurred predominantly with an impact of 0.45 across samples, and some entries at 0.42, without any discernible pattern by strain, substrate, or growth site.

In addition to common pathways, some revealed material-specific traits. Those related to the plaster substrate growth were: glutathione metabolism with an impact in several strains between 0.52 and 0.58, one-carbon pool by folate with a mean impact of 0.24, cysteine and methionine metabolism with a mean of 0.16, and nitrogen metabolism, which appeared only in the metabolism of *K. nitens* PNK013 with an impact of 0.27. Moreover, many pathways in *S. bacillaris* PNK040 showed no statistically significant impact (*p* > 0.05) under laboratory conditions and had the lowest impact values among the others. The only substantial pathway associated solely with this strain was the riboflavin metabolic pathway. In the brick-growth pathway dataset, higher consistency was observed than in the plaster dataset. Stronger brick-linked pathways were purine metabolism with a mean impact of 0.40; glycine, serine, and threonine metabolism with higher impact *ex situ* (mean 0.47) and slightly lower impact in situ (0.28); tyrosine metabolism, which remained high under both growing conditions (mean 0.44); and glycerophospholipid metabolism (mean 0.26). However, under environmental conditions, two specific metabolic signals were identified by pathway analysis: starch and sucrose metabolism, observed in *K. nitens* PNK013 and *S. bacillaris* PNK040 with equal impact (0.40), and the TCA cycle, also observed in *K. nitens* PNK013 with an impact of 0.18.

### Algal-based chemical classes enrichment of substrates

**Fig. 2:**
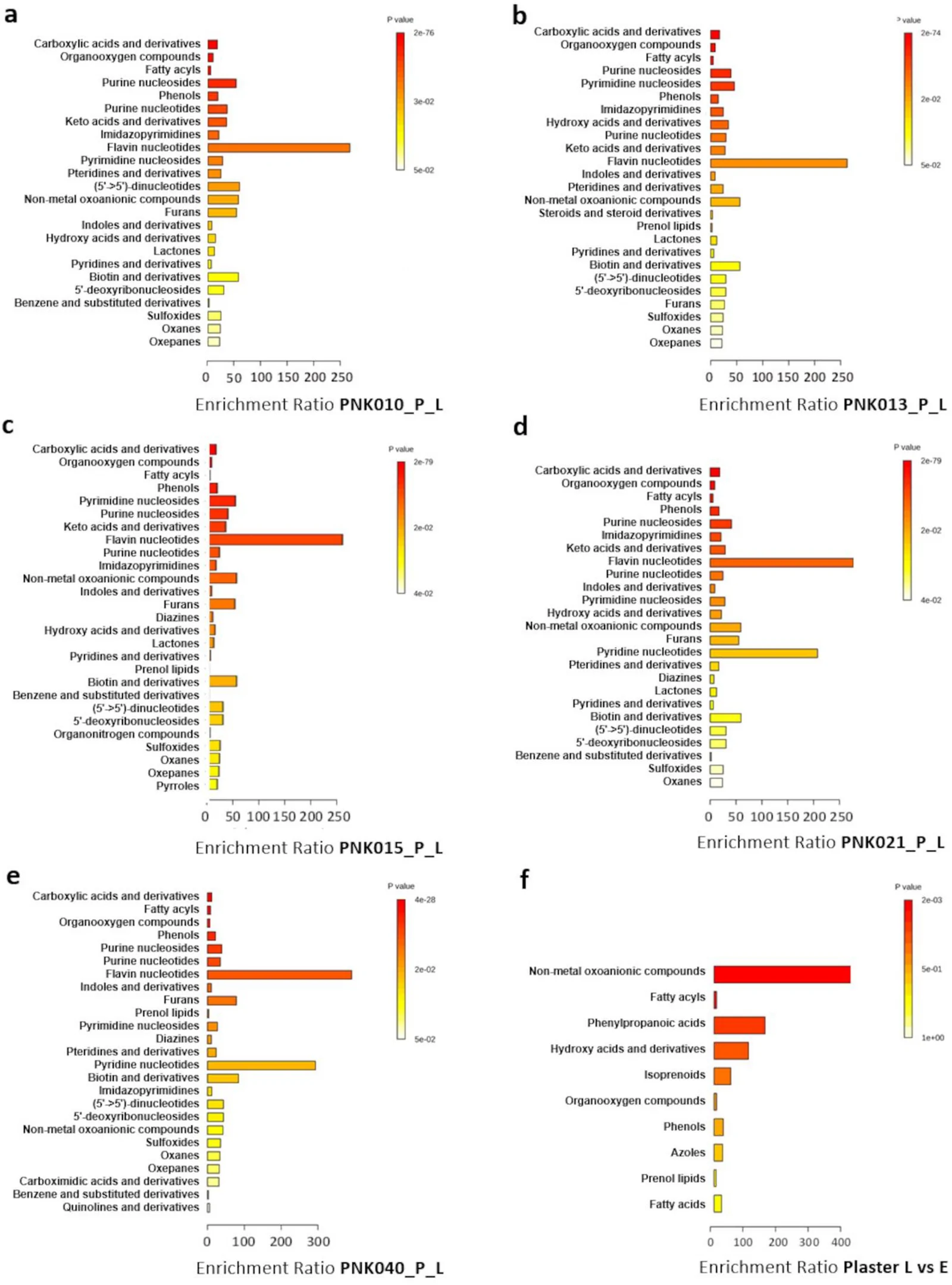
Bar plots of enriched chemical classes obtained from metabolites detected on plasters colonized by algal strains for a year in laboratory conditions; (**a**) plaster (P) overgrown by *Chloroidium saccharophilum* PNK010 in laboratory (L) conditions, (**b**) plaster (P) overgrown by *Klebsormidium nitens* PNK013 in laboratory (L) conditions, (**c**) plaster (P) overgrown by *Bracteacoccus minor* PNK015 in laboratory (L) conditions, (**d**) plaster (P) overgrown by *Diplosphaera chodatii* PNK021 in laboratory (L) conditions, (**e**) plaster (P) overgrown by *Stichococcus bacillaris* PNK040 in laboratory (L) conditions, (**f**) comparison between all algal extracts from plasters kept in laboratory (L) conditions to the ones from the environment (E).

**Fig. 3:**
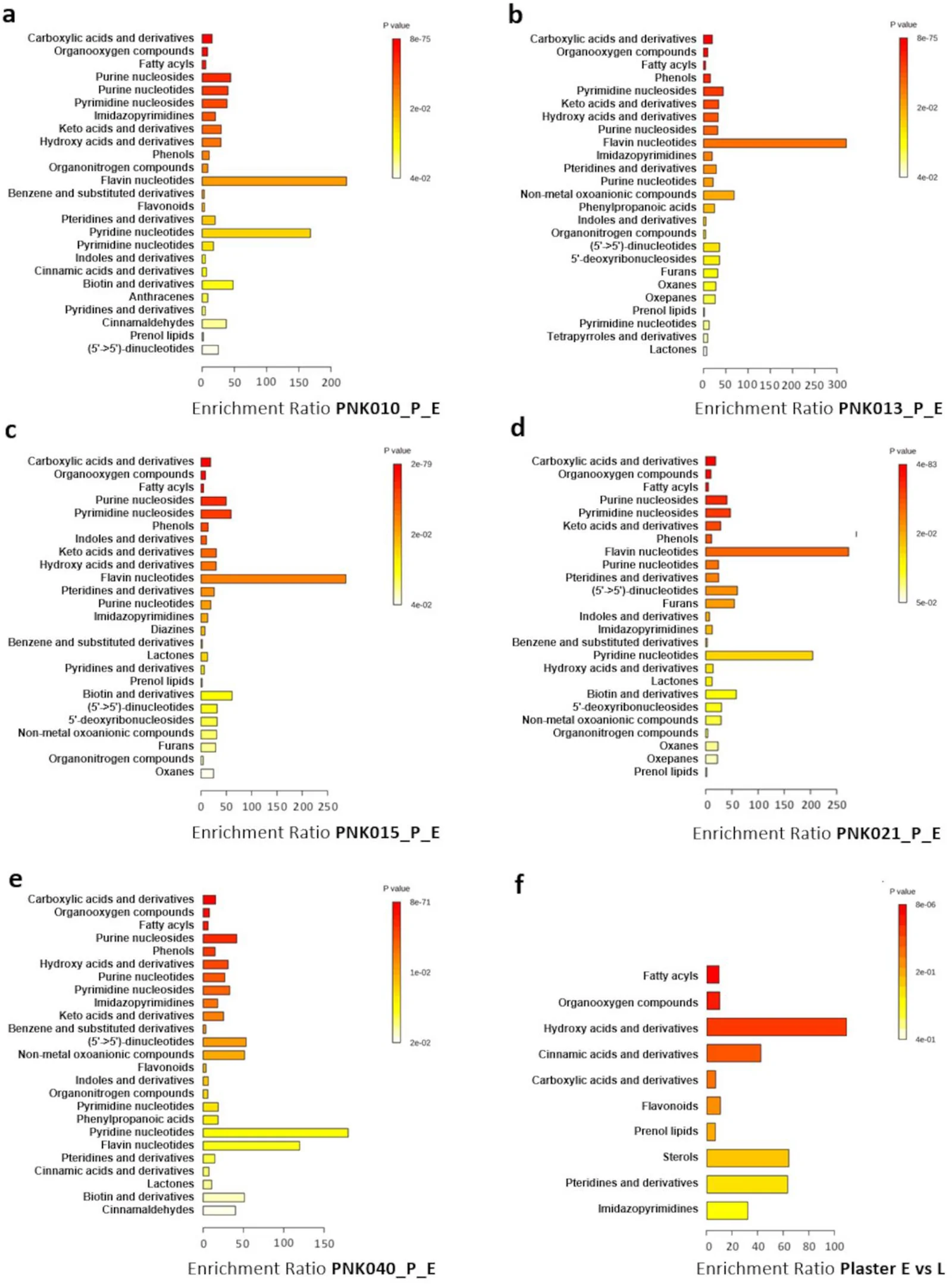
Bar plots of enriched chemical classes obtained from metabolites detected on plasters colonized by algal strains for a year in environmental conditions; (**a**) plaster (P) overgrown by *Chloroidium saccharophilum* PNK010 in the environment (E), (**b**) plaster (P) overgrown by *Klebsormidium nitens* PNK013 in the environment (E), (**c**) plaster (P) overgrown by *Bracteacoccus minor* PNK015 in the environment (E), (**d**) plaster (P) overgrown by *Diplosphaera chodatii* PNK021 in the environment (E), (**e**) plaster (P) overgrown by *Stichococcus bacillaris* PNK040 in the environment (E), (**f**) comparison between all algal extracts from plasters kept in environmental (E) conditions to the ones from the laboratory (L).

**Fig. 4:**
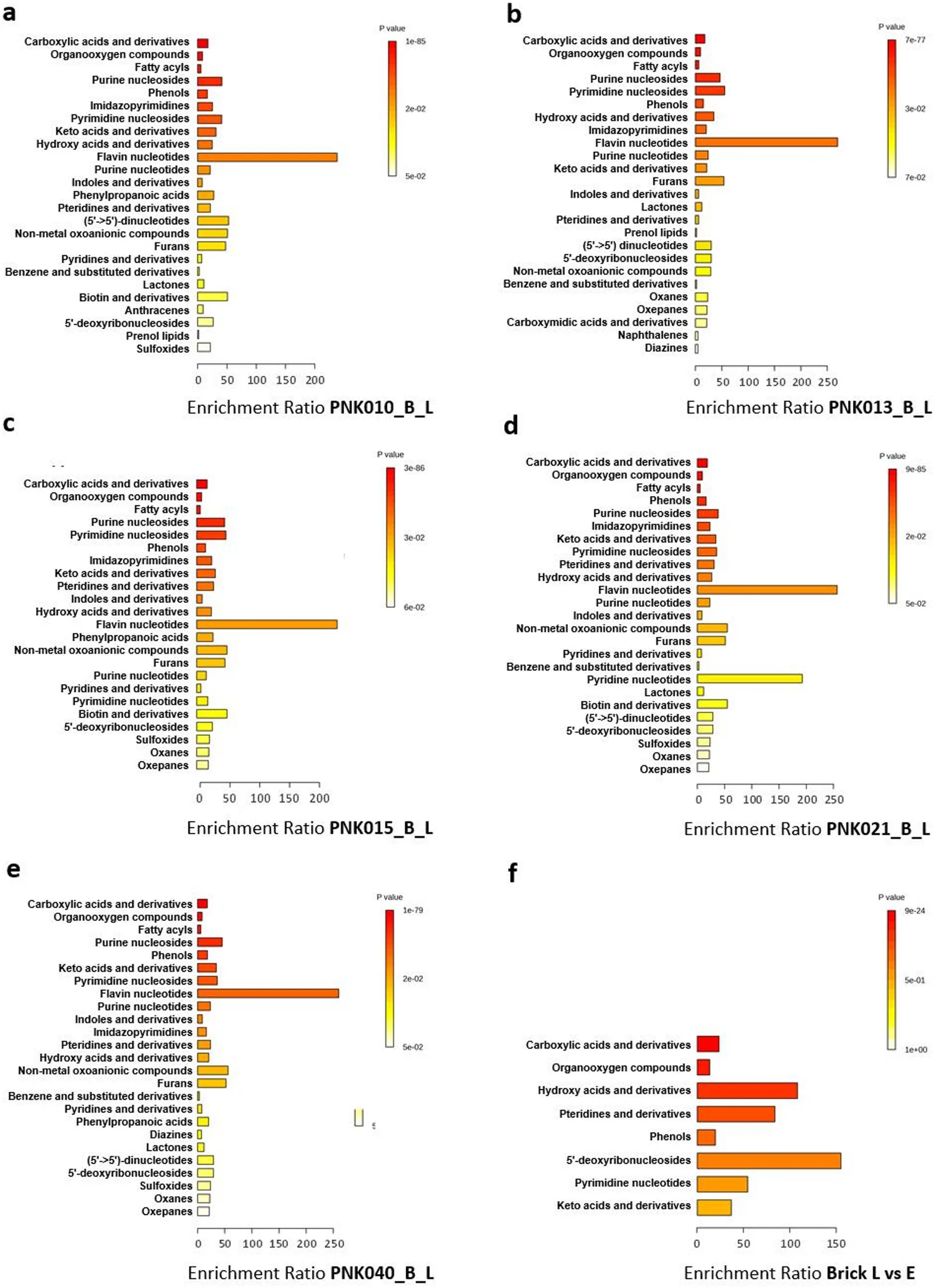
Bar plots of enriched chemical classes obtained from metabolites detected on bricks colonized by algal strains for a year in laboratory conditions; (**a**) brick (B) overgrown by *Chloroidium saccharophilum* PNK010 in laboratory (L) conditions, (**b**) brick (B) overgrown by *Klebsormidium nitens* PNK013 in laboratory (L) conditions, (**c**) brick (B) overgrown by *Bracteacoccus minor* PNK015 in laboratory (L) conditions, (**d**) brick (B) overgrown by *Diplosphaera chodatii* PNK021 in laboratory (L) conditions, (**e**) brick (B) overgrown by *Stichococcus bacillaris* PNK040 in laboratory (L) conditions, (**f**) comparison between all algal extracts from bricks kept in laboratory (L) conditions to the ones from the environment (E).

**Fig. 5:**
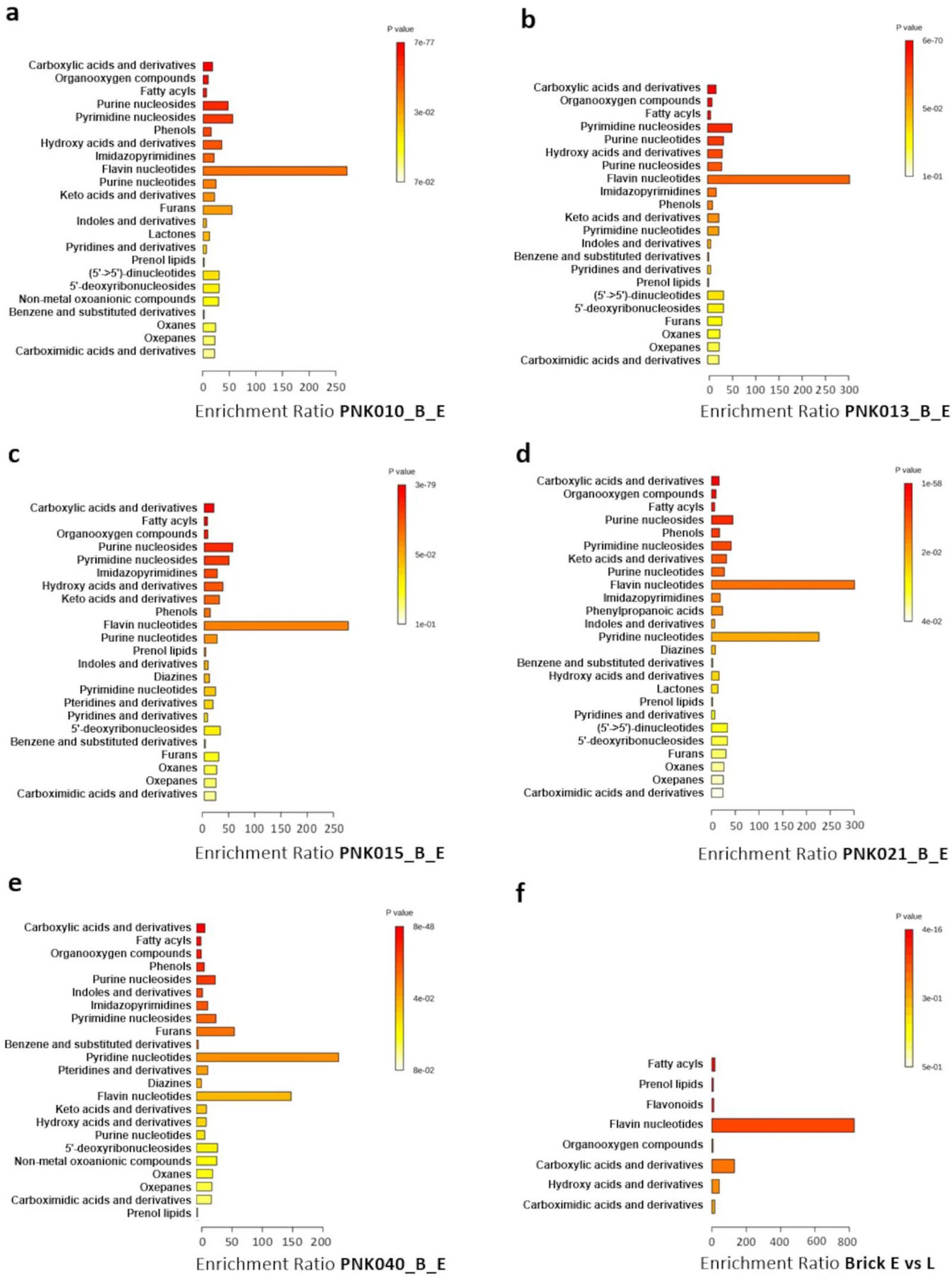
Bar plots of enriched chemical classes obtained from metabolites detected on bricks colonized by algal strains for a year in environmental conditions; (**a**) brick (B) overgrown by *Chloroidium saccharophilum* PNK010 in the environment (E), (**b**) brick (B) overgrown by *Klebsormidium nitens* PNK013 in the environment (E), (**c**) brick (B) overgrown by *Bracteacoccus minor* PNK015 in the environment (E), (**d**) brick (B) overgrown by *Diplosphaera chodatii* PNK021 in the environment (E), (**e**) brick (B) overgrown by *Stichococcus bacillaris* PNK040 in the environment (E), (**f**) comparison between all algal extracts from bricks kept in environmental (E) conditions to the ones from the laboratory (L).

Enrichment analysis identified chemical classes that were overrepresented among metabolites detected in algal-colonized substrates relative to uninoculated controls under both laboratory and environmental conditions. The enrichment results describe metabolite accumulation patterns associated with substrate colonization and biofilm development rather than exclusively the intrinsic metabolic output of algal cells. All of the detected chemical classes in the studied strains are presented in Supplementary Tables S43–S62, while Supplementary Tables S63–S66 present the comparison of all algal-based classes detected on plaster and brick in relation to the growth site. Bar plots of chemical classes enrichment that are presented in Figs. 2, 3, 4 and 5 visualize the enrichment ratio of statistically significant (*p* ≤ 0.05) groups of compounds.

Plaster-associated samples showed consistent enrichment in flavin nucleotides across substrates colonized by all algal strains, both in laboratory and environmental conditions (Figs. 2 and 3). Moreover, the group of carboxylic acids and derivatives, organooxygen compounds, and fatty acyls is relatively stable across strains and growing sites, with the strongest enrichment among the detected chemical classes on plaster (*p* < 0.0001). Pyridine nucleotides in some cases – in the laboratory by the growth of *D. chodatii* PNK021 (Fig. 2:d), while in the environment by *C. saccharophilum* PNK010 (Fig. 3:a), *K. nitens* PNK013 (Fig. 3:b), and *S. bacillaris* PNK040 (Fig. 3:e) also exhibit a high enrichment ratio; however, with less contribution than the previous classes. Across all strains, both *ex situ* and in situ, phenols substantially enriched plaster, whereas in environmental plaster samples, enrichment of benzene derivatives was much more pronounced than in the laboratory (Figs. 2 and 3:a-e). Comparing the whole set of algal-based chemical class enrichments, laboratory-grown biofilms were associated with increased accumulation of non-metal oxoanionic compounds and fatty acyls and phenylpropanoid acids (Fig. 2:f), whereas in the environment, algal biofilms were associated with enrichment of the substrate with fatty acyls, organooxygen compounds, hydroxy acids, and steroids (Fig. 3:f).

In the enrichment of brick substrates, despite the strain and growth conditions, flavin nucleotides predominate in the enrichment ratio (Figs. 4 and 5). Also, the same group of chemical classes as in the case of plaster revealed the highest statistical significance (*p* < 0.0001), but in general, it is less pronounced than in the case of plaster - carboxylic acids and derivatives, organooxygen compounds, and fatty acyls. Brick-associated samples showed comparatively higher enrichment in phenols and phenylpropanoic acids, lactones, prenol lipids, steroids, steroid derivatives, and flavonoids, isoflavonoid compounds than plaster substrates. Comparing the entire set of algal colonization-associated chemical class enrichments between laboratory and environmental growth, there is a substantial difference between the chemical class sets. The laboratory samples are mainly enriched in carboxylic acids and derivatives, hydroxy acids, and keto acids (Fig. 4:f), while the environmental samples are in phenols and phenylpropanoic acids, flavonoids and isoflavonoids, prenol lipids, carboxylic and hydroxy acids (Fig. 5:f).

## Discussion

A larger pool of detected metabolites in algae generally indicates higher metabolic activity and metabolic diversity; however, the number itself does not imply greater functional diversity. Differences in colonization intensity may also contribute to variation in metabolite numbers and signal intensities; therefore, the observed profiles should be interpreted as substrate-associated metabolomic patterns rather than biomass-normalized metabolic rates. The results of metabolite clustering in the studied strains suggest substrate- and condition-associated differences in metabolomic profiles, even when the numbers of metabolites are similar. Enhanced physiological activity and metabolic flexibility enable aerial algae to adjust their biochemistry in response to available nutrients, substrate properties, and external conditions^15^.

The contrasting metabolomic patterns observed on plaster and brick indicate that substrate physicochemical properties appear to contribute to shaping algal metabolism rather than merely providing a growth surface. Plaster and brick differ markedly in mineral composition, porosity, water retention capacity, and alkalinity, all of which can influence nutrient availability, stress exposure, and metabolic regulation in aerophytic algae. Plaster-based substrates are typically rich in calcium sulfate or calcium carbonate and are characterized by relatively high alkalinity and surface reactivity. Such conditions can impose ionic and pH-related stress, potentially triggering broader metabolic reprogramming, including enhanced production of stress-related metabolites^16,17^. The greater heterogeneity of metabolite profiles observed on plaster, particularly under environmental conditions, suggests that this substrate amplifies external fluctuations in moisture availability, temperature, and irradiation. Consequently, algae growing on plaster appear to exhibit greater metabolic plasticity, as evidenced by pronounced shifts in metabolite abundance and stronger differentiation across growing conditions.

The relatively reduced contrast in metabolite abundance patterns and the closer clustering of algal strain samples on the brick indicate a more constrained metabolic response. Brick substrates, composed primarily of silicate-rich minerals with lower intrinsic alkalinity, provide a more stable physical structure than plasters^18^ and offer a relatively predictable microhabitat for microbial colonization. Previous studies have shown that the chemical and mineralogical stability of brick surfaces influences moisture retention and pH buffering, thereby affecting biofilm development and favoring the colonization by aerial algae^12,13^. Brick porosity may facilitate water retention and gradual nutrient exchange, reducing the intensity of abiotic stress signals perceived by algal cells^16^. This stabilization is consistent with the lower metabolic heterogeneity observed across strains and conditions.

The structure and the profile of algal metabolism detected after a year on plaster substrate, under both laboratory and environmental conditions, are consistent with stress-related metabolic adaptation. In most of the strains, metabolic pathways indicate stress-response signaling and robust redox regulation. Glutathione metabolism reflects oxidative stress handling, likely caused by UV radiation, desiccation, or fluctuating nutrients^19^, while cysteine and methionine metabolism, by supporting the production of glutathione, the major intracellular antioxidant, play a role in redox buffering and oxidative stress control^20^. The one-carbon pool supplied by folate facilitates nucleotide synthesis and methylation in response to the external conditions^21^. The isoquinoline alkaloid and alkaloid-like compounds are often linked to stress response and chemical defense^22^; however, the annotation of isoquinoline alkaloid biosynthesis in the present dataset should be interpreted with caution, as it may reflect pathway-map overlap involving shared precursor/intermediate metabolites rather than the presence of a complete functional biosynthetic pathway, while riboflavin metabolism may help in oxidative stress resistance^23^.

The enrichment analysis revealed a consistent dominance of chemical classes associated with primary carbon metabolism, redox processes, and stress adaptation within colonized substrates. However, since the enrichment was calculated relative to uninoculated controls, these patterns should be interpreted as metabolite accumulations associated with algal colonization and biofilm–substrate interactions rather than as direct measures of intracellular algal metabolite production. Furthermore, the analysis confirms that on plaster, flavin nucleotides are highly overrepresented, indicating intense redox activity^24^. Moreover, in the environment, algae shift toward aromatic secondary metabolites (phenylpropanoids, flavonoids). These metabolites support stress-adapted metabolism, likely linked to UV exposure and fluctuating humidity^25,26^.

By contrast, algal metabolism on the brick is more closely related to growth-linked primary metabolism, with carbohydrate enhancement in the environment. A high impact of amino acids metabolic pathways – purine and glycine, serine, and threonine metabolisms indicate active nucleotide turnover/synthesis, while alanine, aspartate, and glutamate metabolism play a role in the central nitrogen and carbon hub—amino acid interconversion, redox balance, and ammonium assimilation^27,28^. Glyoxylate and dicarboxylate metabolism is related to C2 metabolism and stress carbon flux, and is connected to carbon conservation and organic-acid handling^29^. Glycerophospholipid metabolism underpins membrane biogenesis, and shifts in glycerol/glycerophospholipid pathways can co-occur with EPS secretion and cell-surface remodeling under environmental conditions^30^. Moreover, starch and sucrose metabolism, especially in *K. nitens* PNK013 and *S. bacillaris* PNK040 growing in the environment, enables the storage of fixed carbon during brief favorable periods (e.g., wet, illuminated) and remobilizes carbon rapidly upon rehydration^25^. Finally, the TCA cycle, detected only in *K. nitens* PNK013 in situ, integrates carbon catabolism (from sugars, amino acids, and organic acids) and supports energy generation^31^.

The growth and metabolism of the studied strains are not without significance for the stability and durability of the substrates they inhabit. The core pathways detected in this study may affect mineral substrates, potentially lowering their technical resistance and altering their chemical composition. The alanine–aspartate–glutamate metabolic pathway can be accompanied by overflow-like exometabolism release, including extracellular amino acids, N-containing compounds, and low-molecular-weight organic acids. Such metabolites can alter near-surface pH and, through acid attack and ligand complexation (e.g., acetate/oxalate-type ligands), promote Ca^2+^ release and ion mobilization from cementitious/mineral substrates^32–34^. Moreover, the glyoxylate and dicarboxylate metabolic pathway is associated with the production of low-molecular-weight acids, like acetic and citric/isocytric acid. So far, studies on aerial green algae confirm their ability to produce acetate and citrate^12^; however, the glyoxylate cycle also leads to oxalate formation^35^, a direct chemical driver of mineral substrate deterioration^36^. The oxalate-biodeteriorative potential has been widely documented in lichens, driven by fungal activity, whereas the role of algae was once considered indirect, as they supply organic carbon to the fungal partner^37^. However, this study undoubtedly confirms that three of the green algae—*C. saccharophilum* PNK010, *K. nitens* PNK013, and *B. minor* PNK015 – in addition to producing citrate and acetate, produce oxalic acid, which significantly contributes to direct geochemical deterioration. Furthermore, the *D. chodatii* PNK021 is also capable of citrate and oxalate production, while *S. bacillaris* PNK040 only produces isocitrate.

Low-molecular-weight organic acids such as citric/isocitric acid, oxalic acid, and acetic acid, detected in metabolomic profiles, are recognized as important contributors to the biodeterioration of silicate-based building materials, including silicate plasters and clay bricks. Therefore, their interpretation was based on the recurrent occurrence of low-molecular-weight organic acids as a class of compounds rather than on isolated single-compound assignments. In contrast to carbonate substrates, deterioration of aluminosilicates proceeds mainly through ligand-promoted mineral dissolution rather than direct proton-driven attack. Tricarboxylic acids such as citrate and isocitrate, as well as dicarboxylic oxalic acid, efficiently chelate structural and charge-balancing cations (e.g., Al³⁺, Fe³⁺, Ca²⁺, Mg²⁺), thereby enhancing aluminosilicate dissolution and destabilizing the mineral network, which leads to increased porosity and surface weakening^38,39^. Oxalic acid is particularly effective due to its strong complexation capacity and its involvement in microbially mediated mineral weathering and brick alteration processes^40^.

Acetic acid, although a weaker complexing agent, contributes to biodeterioration primarily through localized acidification and mobilization of exchangeable cations, particularly Ca²⁺ present in binders and secondary phases of silicate plasters and bricks. The formation of highly soluble acetate salts facilitates cation leaching and indirectly weakens the mineral matrix, especially under repeated wet–dry cycles^33,38^. When present within algal biofilms, citric/isocitric, oxalic, and acetic acids act in confined microenvironments where low pH and elevated ligand concentrations can be maintained at the material surface, thereby potentially promoting progressive mineral alteration even under near-neutral bulk environmental conditions^40^.

Other carbohydrate metabolism pathways, detected mainly on brick substrates in situ via the metabolism of *K. nitens* PNK013, like starch and sucrose metabolism and citrate cycle (TCA cycle, where citrate production is also a part of the pathway), may also contribute to processes associated with mineral substrate alteration. Enhanced carbohydrate metabolism can support increased synthesis of extracellular polymeric substances (EPS), largely polysaccharidic components of the biofilm matrix^41,42^. On mineral building materials, EPS-rich biofilms can increase near-surface water retention and modify water transport^43,44^, promoting physical deterioration mechanisms associated with wet–dry shrink–swell changes and, where relevant, freeze–thaw action and salt crystallization or efflorescence^38,43^.

The enrichment analysis indicates that organic acids, lipids, and stress-related secondary metabolites were prominent among the enriched chemical classes detected in algal-colonized plaster and brick. Although brick-induced metabolism has been shown to exhibit a growth-induced pattern, environmental aerial algae counteract external land conditions at all times^4^; therefore, the presence of stress-mediated compounds on brick should be unsurprising. An overrepresentation in carboxylic, hydroxy, and keto acids reflects intensive central carbon metabolism and redox balancing during surface-associated growth, potentially contributing to chemical deterioration through acidification, metal chelation, and mineral dissolution of carbonate- and silicate-based materials^38,45^. The consistent presence of fatty acyls and fatty acids indicates membrane remodeling and extracellular lipid production, contributing indirectly to biodeterioration by stabilizing biofilms, enhancing moisture retention, and facilitating the accumulation of acidic metabolites at the material surface^46,47^. Enrichment of phenols, phenylpropanoids, flavonoids, and other aromatic compounds, particularly under environmental conditions, points to stress-adapted secondary metabolism associated with UV protection and oxidative defense; these metabolites are less aggressive chemically but play a significant role in long-term surface alteration through discoloration, redox activity, and persistent organic residue accumulation^48,49^. Non-metal oxoanionic compounds further suggest interactions between biological activity and salt-related physical deterioration processes. Overall, the metabolite profiles indicate a two-tier biodeterioration mechanism for plaster/brick, in which rapid, acid-driven mineral degradation is followed by biofilm-mediated persistence and the chronic accumulation of aesthetic and structural damage.

In the context of particular strains, metabolic pathway analysis, enrichment analysis, and the production of highly deteriorative organic acids enable the definition of the biodeteriorative risk assessment for each algal taxon.

Among the investigated strains, *Chloroidium saccharophilum* PNK010 consistently exhibited the highest overall biodeteriorative risk. Its metabolomic profile was strongly enriched in organic acids under both laboratory and environmental conditions, particularly on brick, indicating a robust capacity for acid-driven chemical weathering. The concurrent presence of active metabolic pathways suggests that this strain not only produces deteriorative metabolites but also maintains prolonged metabolic activity on mineral substrates, thereby intensifying material degradation over time.

*Stichococcus bacillaris* PNK040 also demonstrated a high biodeteriorative potential, particularly on plaster. This contributes to the combined enrichment of organic acids, lipids, and phenolic compounds, which together promote mineral dissolution, biofilm formation, and moisture retention. The confirmed production of citric acid further enhances its chelating capacity, increasing the risk of sustained chemical attack. However, pathway analysis revealed marked sensitivity to environmental conditions, suggesting that PNK040 poses a particularly severe, context-dependent threat of biodeterioration.

In contrast, *Bracteacoccus minor* PNK015 displayed a moderate-to-high biodeteriorative risk characterized by continuous but less extreme chemical activity. The production of acetic, oxalic, and citric acids ensures persistent chemical stress on both plaster and brick, while its relatively conservative pathway profile indicates a steady rather than aggressive metabolic strategy. This pattern suggests that *B. minor* may primarily contribute to long-term, cumulative deterioration rather than to rapid material damage.

*Diplosphaera chodatii* PNK021 represents a distinct biodeterioration strategy dominated by metabolic persistence rather than chemical intensity. High pathway-level activity implies strong adaptive capacity and long-term survival on mineral substrates, whereas enrichment analysis indicates comparatively lower levels of highly aggressive metabolites. As a result, this taxon is likely to induce slow but continuous biodeterioration, driven by sustained colonization and gradual chemical interactions with the substrate.

*Klebsormidium nitens* PNK013 exhibited the highest metabolic versatility and stress-response capacity in pathway analysis; its overall biodeteriorative risk remained moderate. This apparent discrepancy arises from its comparatively low enrichment in strongly deteriorative metabolite classes, suggesting that metabolic adaptability alone does not necessarily translate into high chemical aggressiveness. Moreover, among the studied algae, this taxon exhibited the most conserved metabolomic profile across conditions, which may indicate stronger metabolic homeostasis and/or a greater contribution of constitutive protective mechanisms^50^. This interpretation aligns with the ecology of the *Klebsormidium* genus as a successful aero-terrestrial pioneer with documented tolerance to dehydration and high irradiation, supported by photoprotective capacity, desiccation resilience, and a molecular toolkit associated with terrestrial adaptation^51,52^. Consequently, this species may play a more significant role in biofilm stabilization than in direct material degradation.

The present study should be interpreted within the framework of descriptive metabolomic profiling. Although replicate substrates were included at the colonization stage, the final metabolomic analysis was performed on selected uncontaminated/unialgal samples; therefore, within-group biological variance could not be estimated. Consequently, the observed differences should be interpreted as substrate-associated metabolomic patterns rather than as biologically replicated differential responses. Moreover, when interpreting the enrichment analysis, it should be noted that enrichment was calculated from metabolites detected in colonized substrates relative to uninoculated controls. The resulting chemical classes therefore represent accumulations of colonization-associated metabolites at the biofilm–substrate interface. Accordingly, enriched compounds cannot be unequivocally attributed to direct algal biosynthesis and may also reflect substrate transformation processes or metabolite retention within the biofilm matrix.

Taken together, the data support the interpretation that brick exerts a buffering or constraining effect on algal metabolism, rather than strongly inducing metabolic diversification. Instead, brick appears to moderate environmental stress responses, leading to more conserved metabolic profiles across growing sites. In this context, brick may act as a metabolic stabilizer, dampening rapid or extensive metabolic reprogramming while allowing sustained growth and baseline metabolic activity. Conversely, plaster may function as a metabolic stress amplifier, promoting adaptive metabolic flexibility but also increasing variability among strains and conditions. All of the above contribute to fundamental differences in the biodeterioration mechanisms of plaster and brick. The first undergoes predominantly acid-driven degradation, whereas the latter may experience slower, cumulative deterioration mediated by persistent secondary metabolites and biofilm-growth-associated processes.

## Materials and methods

### Green algal material

**Fig. 6:**
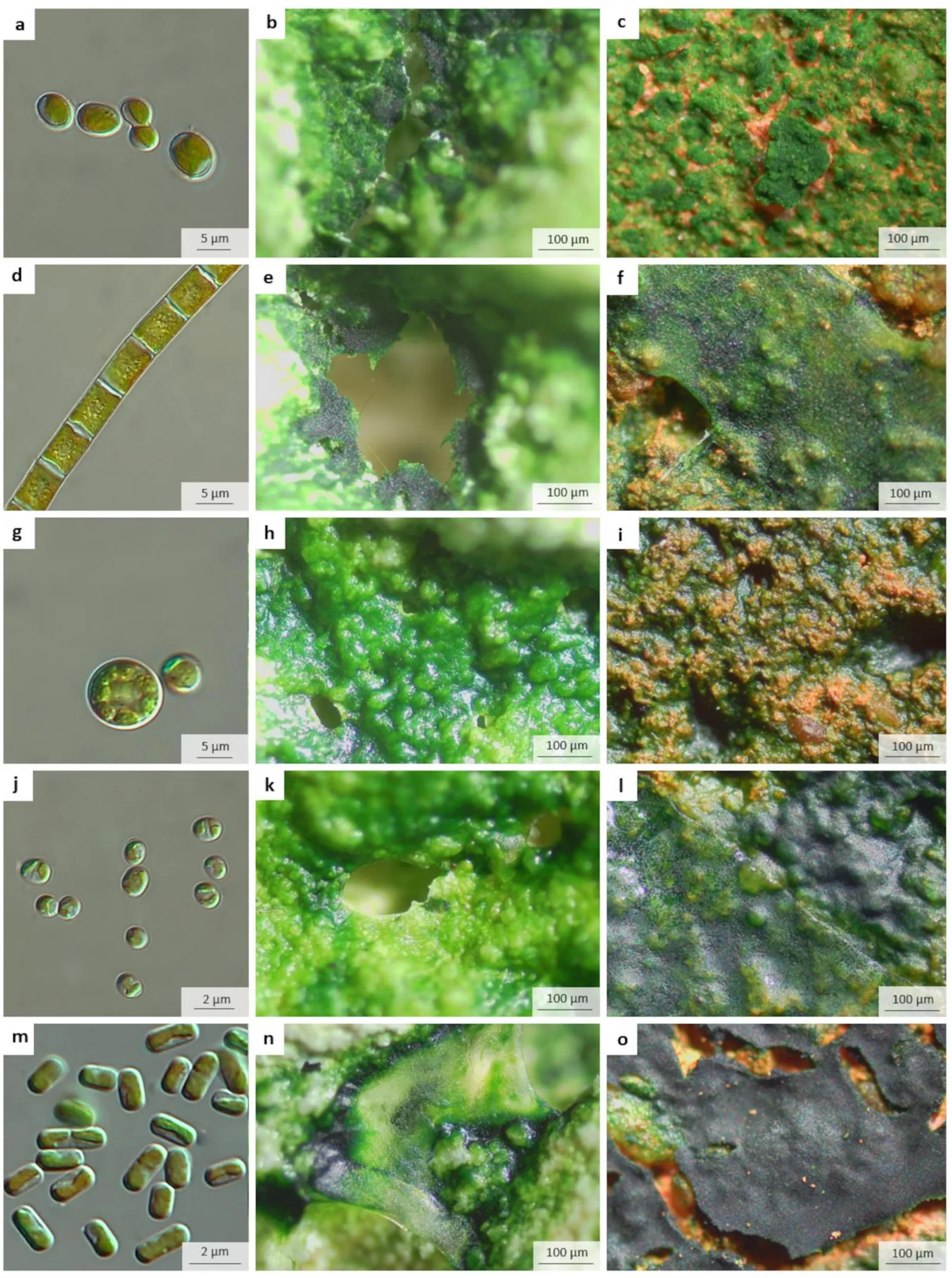
Microphotographs of aerial green algal strains selected for the study; (**a–c**) *Chloroidium saccharophilum* PNK010 (**a**) in a BBM agar culture, (**b**) its biofilm on a plaster, (**c**) its biofilm on a brick; (**d–f**) *Klebsormidium nitens* PNK013 (**d**) in a BBM agar culture, (**e**) its biofilm on a plaster, (**f**) its biofilm on a brick; (**g–i**) *Bracteacoccus minor* PNK015 (**g**) in a BBM agar culture, (**h**) its biofilm on a plaster, (**i**) its biofilm on a brick; (**j–l**) *Diplosphaera chodatii* PNK021 (**j**) in a BBM agar culture, (**k**) its biofilm on a plaster, (**l**) its biofilm on a brick; (**m–o**) *Stichococcus bacillaris* PNK040 (**m**) in a BBM agar culture, (**n**) its biofilm on a plaster, (**o**) its biofilm on a brick.

This research focused on the aerial green algal taxa that are widespread in temperate climates and are known to colonize plaster and brick as pioneer organisms. Green algal strains: *Chloroidium saccharophilum* PNK010, *Klebsormidium nitens* PNK013, *Bracteacoccus mino*r PNK015, *Diplosphaera chodatii* PNK021, and *Stichococcus bacillaris* PNK040 (Fig. 6) were previously isolated from plaster and brick walls of municipal buildings in the city of ca. 645 600 inhabitants in the temperate climate zone, and cultivated under laboratory conditions, as described in^13^. Their taxonomic position was confirmed using traditional and molecular methods, and the strains were evaluated for their potential for geophysical biodeterioration^13^. Therefore, these strains have been selected for further metabolomic-based research.

### Experiment design

The algal strains were inoculated onto sterilized experimental substrates – polysilicate plaster and red brick as unialgal cultures as described in^13^ and cultivated for 12 months, both in laboratory and environmental conditions, along with the control samples. Each strain was inoculated onto three replicate substrates, and one substrate was left uninoculated as the control (in total, 40 plaster and 40 brick samples were used). In the laboratory, substrates were maintained vertically in plastic containers, with ventilation pores sealed with a micropore membrane to prevent external contamination. Proper humidity was maintained by spraying the substrates every three weeks with a small amount of sterilized tap water under sterile conditions, and cultivation was performed under the same conditions as the strains were grown in agar cultures. In situ, samples were mounted on the experimental frame in vertical series, with each series corresponding to a strain transect and spaced 1.5 m apart, in an open space 20 m from any natural spots with visible phototrophic colonization. Within each strain transect, the uppermost position was occupied by the control sample, followed by three replicate strain samples arranged downstream. This configuration minimized the risk of water runoff along the series contaminating the control sample during precipitation events. The environmental samples were left unmanipulated from one spring season to the next.

Prior to the metabolomic assay, experimental samples were checked for possible contamination using a Nikon Eclipse 50i Light Microscope (Precoptic Co., Warsaw, Poland). Samples were observed as biofilms on substrates at 100x magnification with external lighting, and on microscopic slides at 600x magnification, by taking a small portion of the biofilms from the substrates using a sterile soft brush. Since *ex situ* samples were maintained uncontaminated, one randomly selected substrate per strain was selected for study. Among environmental substrates, those that maintained unialgal were the first in the line after the control in strain transects; therefore, they were selected for metabolomic assay. Consequently, the metabolomic dataset was interpreted as a descriptive profile of selected uncontaminated/unialgal biofilm samples rather than as a fully replicated quantitative comparison. This selection minimized metabolomic interference from non-target organisms, particularly in environmentally exposed samples.

### Preparation of samples for metabolomic assay

The algal biomass was separated from the experimental substrates as follows. Each sample was placed in a plastic Petri dish with the inoculated surface facing down. To each dish, LC-MS-grade water was added, with the volume normalized to the inoculated surface area. Partially submerged samples were placed in an ultrasonic bath (Branson 1510 Ultrasonic Cleaner, Branson Ultrasonics, USA) for 3 min to facilitate biomass separation. Afterward, the suspended biomass was collected and transferred to Eppendorf tubes. This procedure was repeated using 5 ml of LC-MS-grade water and an ultrasonic bath for 1 min. Collected samples were centrifuged for 10 min at 6000 rpm, and the supernatants were discarded. The supernatants were collected carefully, ensuring no visible liquid remained above the biomass. Algal biomass was then weighed, and the following extraction procedure was scaled up or down based on biomass weight. To each sample of 10 mg biomass weight, 250 µl of LC-MS-grade water and 750 µl of acetone were added, after which the samples were left to incubate at room temperature for 24 h. Subsequently, homogenization was performed using 3 steel beads per sample in a TissueLyser II homogenisator (QIAGEN, Hilden, Germany). Homogenization time was set to 1 min, and the procedure was repeated 3 times, with a 30-second break between cycles. Homogenized samples were incubated for 30 min at − 20 °C. Afterward, the extracts were centrifuged for 5 min at 11000 rpm, and the supernatant was transferred to new, pre-weighed Eppendorf tubes. Samples were dried for 24 h using a speed-vac type apparatus under 1300 rpm and 2 × 10^− 3^ mbar vacuum, and then weighed again. Dried extracts were suspended in 120 µl of methanol and briefly vortexed. To facilitate dissolution, samples were sonicated for a few seconds and then centrifuged for 5 min at 12000 rpm using a mySPIN™ 12 Mini Centrifuge (Thermo Fisher Scientific, USA). Supernatants in a volume of 80 µl were transferred to glass inserts compatible with standard HPLC vials.

### Metabolomic analysis

The LC-MS/MS analysis was carried out on the Bruker Elute UHPLC system with Hystar 3.3 software and an ultrahigh resolution 60000 + mass spectrometer Bruker Impact II (Bruker Daltonics GmbH, Germany), ESI QTOF-MS, equipped with Metaboscape ver. 2022b and Data Analysis 4.2 ver. 2022b (Bruker Daltonics GmbH, Germany) software. For the procedure, a Bruker 100 × 2.1 mm, C-18 UHPLC Intensity Solo Column was used. LC-MS-grade water with 0.1% HCOOH and acetonitrile with 0.1% HCOOH were applied as eluent A and B, respectively. Chromatography was performed using eluent gradient, with the following parameters: 0 min and 2 min − 99% A, 17 min − 1% A, 20 min − 1% A, 20.1 min, 22 min, 30 min − 99% A; flow was at a level of 0.25 µlmin^− 1^ from 0 min to 20 min and 0.35 µlmin^−1^ from 20.1 min to 30 min. A constant temperature of 40 °C was maintained for the UHPLC column and 4 °C for the autosampler. For the injection, a volume of 5 µl was used. Auto MS/MS operated at the *m/z* range of 50–1200. Collision-Induced Dissociation (CID) used an absolute area threshold of 5000 counts; active exclusion of 2 spectra; release after 0.3 min, isolation mass: for *m/z* = 100, width was set to 4, for *m/z* = 300, width was 5, for *m/z* = 500, width was 6, while for *m/z* = 1000, width was set to 8; collision energy value was 30 eV. Internal calibration of 10 mM sodium formate (water to isopropanol, 1:1 v/v) ions was performed automatically in MetaboScape ver. 2022b with the use of a syringe pump at an infusion flow rate of 0.12 mL h^−1^, using a high precision calibration (HPC) mode. Each sample was measured in both positive- and negative-ion modes. The untargeted annotations were performed using MetaboScape ver. 2022b mass deviation (Δ*m/z*) criterion under 3 ppm and mSigma value under 20 as the maximum acceptable deviation of the compound mass and the isotopic pattern, respectively. All the molecular formulas were obtained using the Smart Formula tool and the C, H, N, O, P, S, Cl, Br, I, and F elements. MS/MS spectra were automatically matched against MS/MS libraries, i.e., Bruker HMDB 2.0 library, Bruker Plant Library, MassBank of North America (MoNA) library (Mass Bank of North America, 2024), and NIST ver. 2020 MSMS library (Mass Spectrometry Data Center, 2022). Thus, metabolite assignments were evaluated using a multi-parameter workflow that included accurate mass, mass error, isotopic pattern fit, retention time information where available, and MS/MS spectral similarity; the corresponding annotation parameters are reported for individual compounds in Supplementary Table S1.

### Preparation of metabolic data

To distinguish metabolites associated with algal colonization from compounds originating from the substrate (plaster or brick), each strain was directly compared with its corresponding control sample. A metabolite was retained for analysis if its abundance in at least one strain exceeded that in the control (fold change, FC > 2). This criterion accounts for biological variability among strains, as individual strains may produce unique metabolites; therefore, even a single pronounced increase relative to the control was considered indicative of a potential colonization-associated origin. Metabolites were removed only when none of the strains exhibited elevated abundance relative to the control and their abundance was consistently higher across most control samples. Such metabolites were considered background signals arising from the substrate and were excluded from further interpretation. Only metabolites with FC > 2 were included in downstream analyses, as these values indicate a clearly higher abundance in experimental samples compared to controls. Thus, the retained metabolites were interpreted as colonization-associated relative to the corresponding uninoculated substrate controls, rather than as metabolites unequivocally induced by substrate interaction. These metabolites were subsequently used for exploratory analyses of metabolic pathways and compound classes. Raw metabolomic data detected in positive and negative ion modes were submitted to the figshare online database (https://doi.org/10.6084/m9.figshare.31323610), while a set of processed metabolic data is included in the Supplementary Tables S1–S21.

### Metabolomic data processing and analysis

All metabolite datasets exported using Metaboscape ver. 2022b were examined utilizing MetaboAnalyst 6.0^53,54^. Prior to statistical evaluation, the data were log-transformed and auto-scaled. Detected metabolites were annotated with the KEGG *Chlorella variabilis* reference pathway database^14^. Metabolic pathways were considered significantly overrepresented among retained annotated metabolites based on adjusted *p*-values (*p* ≤ 0.05), whereas the topology analysis was used to estimate pathway impact scores. The set of detected metabolic pathways is provided in Supplementary Tables S22–S42. Moreover, unsupervised multivariate analysis was performed using Principal Component Analysis (PCA) to assess overall clustering patterns, and heatmaps of metabolic profiles were used to reveal global differences between samples. Chemical class enrichment analysis was carried out using the main-class metabolite set library to identify chemical categories overrepresented in each comparison. The enrichment ratio was calculated as the proportion of detected metabolites belonging to a given class relative to the expected background frequency. Thus, chemical-class enrichment reflects numerical overrepresentation among detected metabolites and should not be interpreted as a direct measure of metabolite concentration or summed abundance. To identify metabolites enriched or reduced relative to the corresponding control, fold-change (FC) analysis was applied, using thresholds of FC > 2 (increased) or FC < 0.5 (decreased). The set of chemical classes of metabolites detected is included in Supplementary Tables S43–S66. Because the final metabolomic profiling was based on one selected substrate per strain–substrate–condition combination, the analyses were not interpreted as biological replicate-based differential abundance testing. Accordingly,* p*-values reported for pathway and chemical-class analyses refer to overrepresentation statistics rather than to within-group biological variance. Chemical classes and pathway annotations with *p*-values ≤ 0.05 were considered statistically overrepresented.

## Supplementary Information

Below is the link to the electronic supplementary material.

Supplementary Material 1

Supplementary Material 2

Supplementary Material 3

Supplementary Material 4

Supplementary Material 5

## Data Availability

The datasets generated and analysed during the current study are available in the figshare database (https://doi.org/10.6084/m9.figshare.31323610), and are included in this article (and its Supplementary Information files).

## References

[1] Allsopp, D., Seal, K. J. & Gaylarde, C. C. *Introduction to Biodeterioration, *II ed, 1–10 (Cambridge University Press, 2004).

[2] Sterflinger, K. & Piňar, G. Microbial deterioration of cultural heritage and works of art—tilting at windmills? *Appl. Microbiol. Biotechnol.* **97**, 9637–9646. 10.1007/s00253-013-5283-1 (2013). 24100684 PMC3825568

[3] Samad, L. K. & Adhikary, S. P. Diversity of micro-algae and cyanobacteria on building facades and monuments in India. *Algae* **23**, 91–114. (2008). 10.4490/algae.2008.23.2.091 (2008).

[4] Nowicka-Krawczyk, P., Komar, M. & Gutarowska, B. Towards understanding the link between the deterioration of building materials and the nature of aerophytic green algae. *Sci. Total Env*. **802**, 149856. 10.1016/j.scitotenv.2021.149856 (2022). 34454144

[5] Sharma, N. K. *Airborne Algae: Their Significance* 1–103 (Nova Science Publisher, 2015).

[6] Leadbeater, B. S. C., & Callow, M. E. Formation Composition and Physiology of Algal Biofilms. in *Biofilms-Science and Technology* (eds Melo, L. F., Bott, T. R., Fletcher, M. & Capteville, D.) 149–162 (Kluwer Academic, 1992).

[7] Grbić, M. L., Vukojević, J., Simić, G. S., Krizmanić, J. & Stupar, M. Biofilm forming cyanobacteria, algae and fungi on two historic monuments in Belgrade. *Serbia Arch. Biol. Sci. Belgrade*. **62**, 625–631. 10.2298/ABS1003625L (2010).

[8] Quagliarini, E. et al. Effect of temperature and relative humidity on algae biofouling on different fired brick surfaces. *Constr. Build. Mater.* **199**, 396–405. 10.1016/j.conbuildmat.2018.12.023 (2019).

[9] Barberousse, H., Rout, B., Yéprémian, C. & Boulon, G. An assessment of facade coatings against colonisation by aerial algae and cyanobacteria. *Build. Environ.* **42**, 2555–2561. 10.1016/j.buildenv.2006.07.031 (2007).

[10] D’Orazio, M. et al. Effects of water absorption and surface roughness on the bioreceptivity of ETICS compared to clay bricks. *Build. Environ.* **77**, 20–28. 10.1016/j.buildenv.2014.03.018 (2014).

[11] Guillitte, O. & Dreesen, R. Laboratory chamber studies and petrographical analysis as bioreceptivity assessment tools of building materials. *Sci. Total Environ.* **167**, 365–374. 10.1016/0048-9697(95)04596-S (1995).

[12] Komar, M. et al. Biodeterioration potential of algae on building materials-model study. *Int. Biodeterior. Biodegrad*. **180**, 105593. (2023). 10.1016/j.ibiod.2023.105593 (2023).

[13] Nowicka-Krawczyk, P. Growth strategy of aerial green algae on building materials in the temperate climate zone and its relevance to substrate biodeterioration. *Sci. Rep.* **16**, 2167. 10.1038/s41598-025-31926-x (2026). PMC12808225 41372395

[14] Kanehisa, M., Furumichi, M., Sato, Y., Matsuura, Y. & Ishiguro-Watanabe, M. KEGG: biological systems database as a model of the real world. *Nucleic Acids Res.* **53**, D672–D677. 10.1093/nar/gkae909 (2025). 39417505 PMC11701520

[15] Nwokorogu, V. C. & Sabiu, S. Targeted and Untargeted Metabolomics for Algal Characterization and Application in Algae and Algal Metabolites. in R*eference Series in Phytochemistry* (eds Harish, M. & Sabiu) 1–32 (Springer, 2025). 10.1007/978-3-031-80866-1_34-1.

[16] Pinna, D. Microbial Growth and its Effects on Inorganic Heritage Materials. in *Microorganisms in the Deterioration and Preservation of Cultural Heritage* (ed Joseph, E.) (Springer, 2021). 10.1007/978-3-030-69411-1_1.

[17] Chen, X., Termonia, P. & Hamdi, R. Van Den Bossche, N. Modeling algae growth on masonry in hygrothermal simulations: developing a new response indicator. *Build. Environ.* **269**, 1–15. 10.1016/j.buildenv.2024.112437 (2025).

[18] Chapagain, Y. P. et al. A case study on mineralogy and physico-mechanical properties of commercial bricks produced in Nepal. *SN Appl. Sci.* **2**, 1856. 10.1007/s42452-020-03535-y (2020).

[19] Koletti, A., Skliros, D., Dervisi, I., Roussis, A. & Flemetakis, E. Oxidative stress responses in microalgae: Modern insights into an old topic. *Appl. Microbiol.* **5**, 37. (2025). 10.3390/applmicrobiol5020037 (2025).

[20] Diaz-Vivancos, P., de Simone, A., Kiddle, G. & Foyer, C. H. Glutathione-linking cell proliferation to oxidative stress. *Free Radic Biol. Med.* **89**, 1154–1164. (2015). 10.1016/j.freeradbiomed.2015.09.023 (2015). 26546102

[21] Holzinger, A. et al. Transcriptomics of desiccation tolerance in the streptophyte green alga *Klebsormidium* Reveal a land plant-like defense reaction. *PLoS ONE*. **9**, e110630. (2014). 10.1371/journal.pone.0110630 (2014). 25340847 PMC4207709

[22] Dey, P. et al. Analysis of alkaloids (indole alkaloids, isoquinoline alkaloids, tropane alkaloids). in *Recent Advances in Natural Products Analysis* (eds Silva, A. S., Nabavi, S. F., Saeedi, M & Nabavi, S. M.) 505–567 (Elsevier, 2020). 10.1016/B978-0-12-816455-6.

[23] Ashoori, M. & Saedisomeolia, A. Riboflavin (vitamin B₂) and oxidative stress: A review. *Br. J. Nutr.* **111**, 1985–1991. (2014). 10.1017/S0007114514000178 (2014). 24650639

[24] Massey, V. The chemical and biological versatility of riboflavin. *Biochem. Soc. Trans.* **28**, 283–296 (2000). 10961912

[25] Holzinger, A. & Karsten, U. Desiccation stress and tolerance in green algae: Consequences for ultrastructure, physiological and molecular mechanisms. *Front. Plant. Sci.* **4**, 327. (2013). 10.3389/fpls.2013.00327 (2013). 23986769 PMC3749462

[26] De Vries, S. et al. The evolution of the phenylpropanoid pathway entailed pronounced radiations and divergences of enzyme families. *Plant. J.* **107**, 975–1002. 10.1111/tpj.15387 (2021). 34165823

[27] Han, M. et al. l-Aspartate: An essential metabolite for plant growth and stress acclimation. *Molecules* **26**, 1887. (2021). 10.3390/molecules26071887 (2021). 33810495 PMC8037285

[28] Fortunato, S. et al. The role of glutamine synthetase (GS) and glutamate synthase (GOGAT) in the improvement of nitrogen use efficiency in cereals. *Biomolecules* **13**, 1771. (2023). 10.3390/biom13121771 (2023). 38136642 PMC10742212

[29] Dellero, Y., Jossier, M., Schmitz, J., Maurino, V. G. & Hodges, M. Photorespiratory glycolate–glyoxylate metabolism. *J. Exp. Bot.* **67**, 3041–3052. 10.1093/jxb/erw090 (2016). 26994478

[30] Khozin-Goldberg, I. & Cohen, Z. Unraveling algal lipid metabolism: Recent advances in gene identification. *Biochimie* **93**, 91–100. 10.1016/j.biochi.2010.07.020 (2011). 20709142

[31] Owen, O. E., Kalhan, S. C. & Hanson, R. W. The key role of anaplerosis and cataplerosis for citric acid cycle function. *J. Biol. Chem.* **277**, 30409–30412. (2002). 10.1074/jbc.R200006200 (2002). 12087111

[32] Paczia, N. et al. Extensive exometabolome analysis reveals extended overflow metabolism in various microorganisms. *Microb. Cell. Fact.* **11**, 122. 10.1186/1475-2859-11-122 (2012). 22963408 PMC3526501

[33] Bertron, A. Understanding interactions between cementitious materials and microorganisms: a key to sustainable and safe concrete structures in various contexts. *Mater. Struct.* **47**, 1787–1806. 10.1617/s11527-014-0433-1 (2014).

[34] Negi, A. & Sarethy, I. P. Microbial biodeterioration of cultural heritage: Events, colonization, and analyses. *Microb. Ecol.* **78**, 1014–1029 (2019). https://www.jstor.org/stable/48701594 31025063 10.1007/s00248-019-01366-y

[35] Yoon, J-J., Hattori, T. & Shimada, M. A metabolic role of the glyoxylate and tricarboxylic acid cycles for development of the copper-tolerant brown-rot fungus *Fomitopsis palustris*. *FEMS Microbiol. Lett.* **217**, 9–14. (2002). 10.1016/S0378-1097(02)01001-7 (2002). 12445639

[36] De Windt, L. & Devillers, P. Modeling the degradation of Portland cement pastes by biogenic organic acids. *Cem. Concr Res.* **40**, 1165–1174. 10.1016/j.cemconres.2010.03.005 (2010).

[37] Adamo, P. & Violante, P. Weathering of rocks and neogenesis of minerals associated with lichen activity. *Appl. Clay Sci.* **16**, 229–256. 10.1016/S0169-1317(99)00056-3 (2000).

[38] Warscheid, T. & Braams, J. Biodeterioration of stone: A review. *Int. Biodeterior. Biodegrad*. **46**, 343–368. (2000). 10.1016/S0964-8305(00)00109-8 (2000).

[39] Cama, J. & Ganor, J. The effects of organic acids on the dissolution of silicate minerals: A case study of oxalate catalysis of kaolinite dissolution. *Geochim. Cosmochim. Acta*. **70**, 2191–2209. 10.1016/j.gca.2006.01.028 (2006).

[40] Gadd, G. M. Metals, minerals and microbes: geomicrobiology and bioremediation. *Microbiol* **156**, 609–643. 10.1099/mic.0.037143-0 (2010). 20019082

[41] Rossi, F. & De Philippis, R. Role of cyanobacterial exopolysaccharides in phototrophic biofilms and biological soil crusts. *Life* **5**, 1218–1238. 10.3390/life5021218 (2015). 25837843 PMC4500136

[42] Das, T. Surajit Genetic regulation, biosynthesis and applications of extracellular polysaccharides of the biofilm matrix of bacteria. *Carbohydr. Polym.* **291**, 119536. 10.1016/j.carbpol.2022.119536 (2022). 35698329

[43] Gaylarde, C. & Baptista-Neto, J. A. Microbiologically induced aesthetic and structural changes to dimension stone. *npj Mater. Degrad.* **5**, 33. 10.1038/s41529-021-00180-7 (2021).

[44] Schröer, L., De Kock, T., Godts, S., Boon, N. & Cnudde, V. The effects of cyanobacterial biofilms on water transport and retention of natural building stones. *Earth Surf. Process. Landf.* **47**, 2234–2249. 10.1002/esp.5355 (2022).

[45] Gorbushina, A. A. Life on the rocks. *Environ. Microbiol.* **9**, 1613–1631. 10.1111/j.1462-2920.2007.01301.x (2007). 17564597

[46] Crispim, C. A., Gaylarde, C. C. & Gaylarde, P. M. Biofilms on church walls in Porto Alegre, RS, Brazil, with special attention to cyanobacteria. *Int. Biodeterior. Biodegrad*. **54**, 121–124. 10.1016/j.ibiod.2004.03.001 (2004).

[47] De Muynck, W., De Belie, N. & Verstraete, W. Microbial carbonate precipitation in construction materials. *Ecol. Eng.* **36**, 118–136. 10.1016/j.ecoleng.2009.02.006 (2009).

[48] Saiz-Jimenez, C. Biogeochemistry of weathering processes in monuments. *Geomicrobiol. J.* **16**, 27–37. 10.1080/014904599270721 (1999).

[49] Gadd, G. M. Geomycology: biogeochemical transformations of rocks and minerals. *Mycol. Res.* **121**, 1–20. 10.1016/j.mycres.2006.12.001 (2017). 17307120

[50] Kusano, M. et al. Unbiased characterization of genotype-dependent metabolic regulations by metabolomic approach in *Arabidopsis thaliana*. *BMC Syst. Biol.* **1**, 53. (2007). 10.1186/1752-0509-1-53 (2007). 18028551 PMC2233643

[51] Hori, K. et al. *Klebsormidium flaccidum* genome reveals primary factors for plant terrestrial adaptation. *Nat. Commun.* **5**, 3978. 10.1038/ncomms4978 (2014). 24865297 PMC4052687

[52] Holzinger, A. & Pichrtová, M. Abiotic stress tolerance of charophyte green algae: New challenges for omics techniques. *Front. Plant. Sci.* **7**, 678. (2016). 10.3389/fpls.2016.00678 (2016). 27242877 PMC4873514

[53] Pang, Z. et al. MetaboAnalyst 6.0: towards a unified platform for metabolomics data processing, analysis and interpretation. *Nucleic Acids Res.* **52**, W398–W406. 10.1093/nar/gkae253 (2024). 38587201 PMC11223798

[54] Xia, J., Wishart, D. S. & Using MetaboAnalyst 3.0 for comprehensive metabolomics data analysis. *Curr. Protoc. Bioinf.* **55**, 14.10.1–14.10.91 (2016). 10.1002/cpbi.11 27603023

