# Supplementary material for: Assessing the potential for biogeochemical deterioration of building materials by green algae in temperate climate via an integrated metabolomic approach
Source: Sci Rep. 2026 Jun 19;16:28186. doi: 10.1038/s41598-026-58452-8 (PMC13554127; doi:10.1038/s41598-026-58452-8)
Supplement: Supplementary file 1 [file 41598_2026_58452_MOESM1_ESM.pdf]

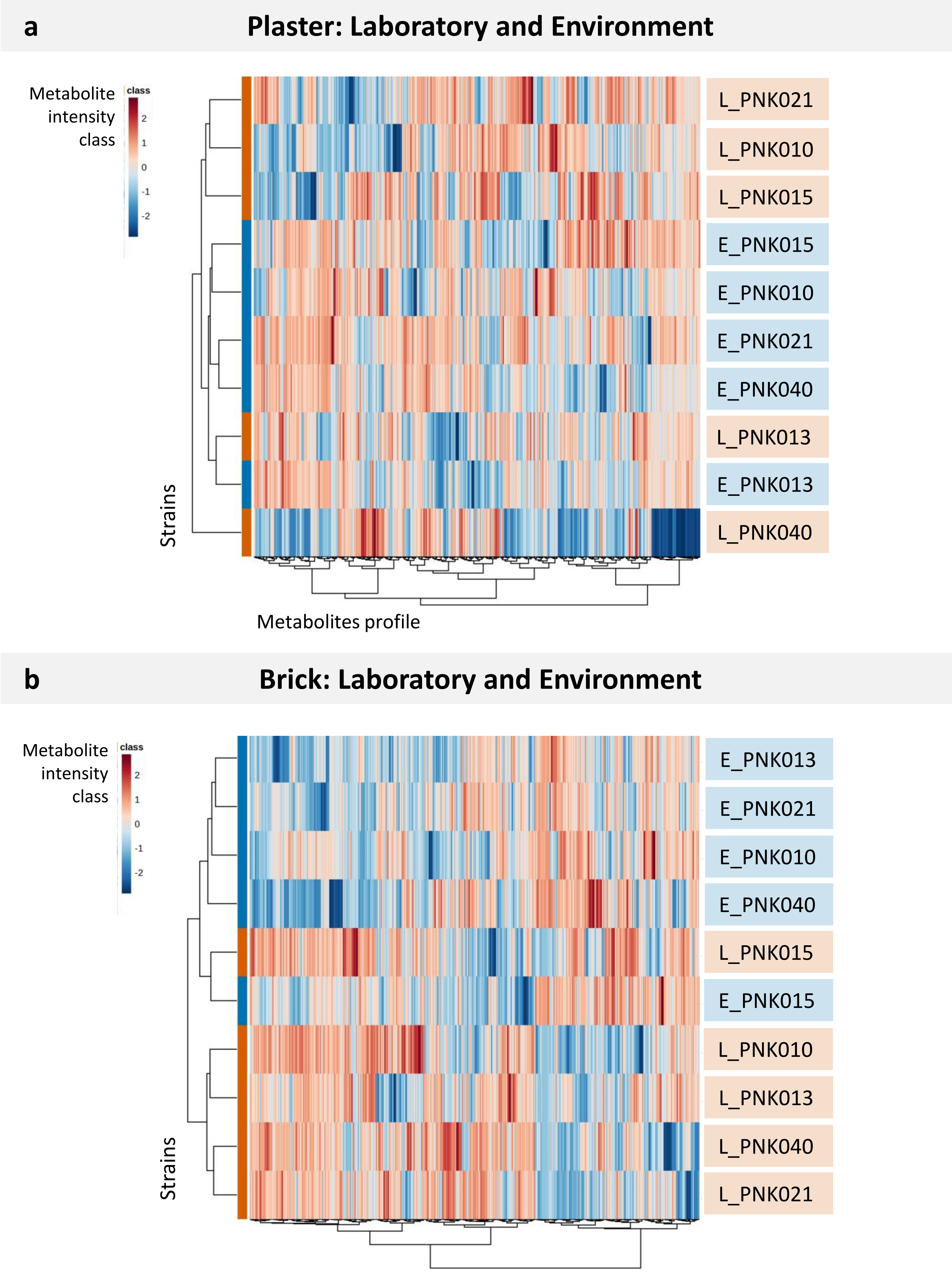

Figure S1. Heatmap of metabolomic profiles for algal strains growing for a year on (**a**) plaster and (**b**) brick in relation to the growing site: laboratory (L) vs. environment (E); hierarchical clustering was based on normalized metabolite intensities, the color intensity represents metabolite abundance (scaled values), and the strains refer to the algal taxa: (PNK010*) Chloroidium saccharophilum*, (PNK013) *Klebsormidium nitens*, (PNK015) *Bracteacoccus minor*, (PNK021) *Diplosphaera chodatii*, (PNK040) *Stichocccus bacillaris*.

a **Laboratory: Plaster and Brick**

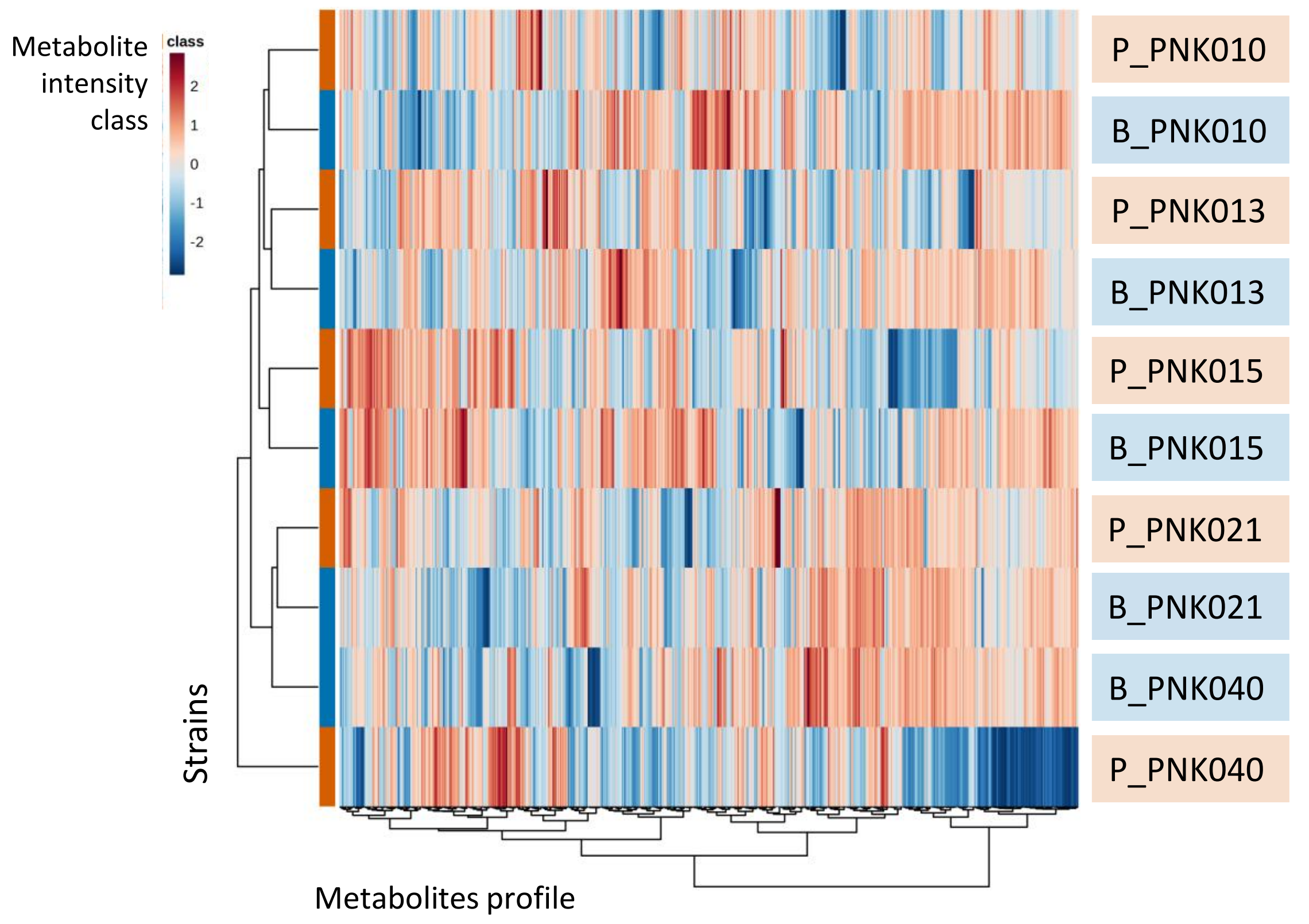

b **Environment: Plaster and Brick**

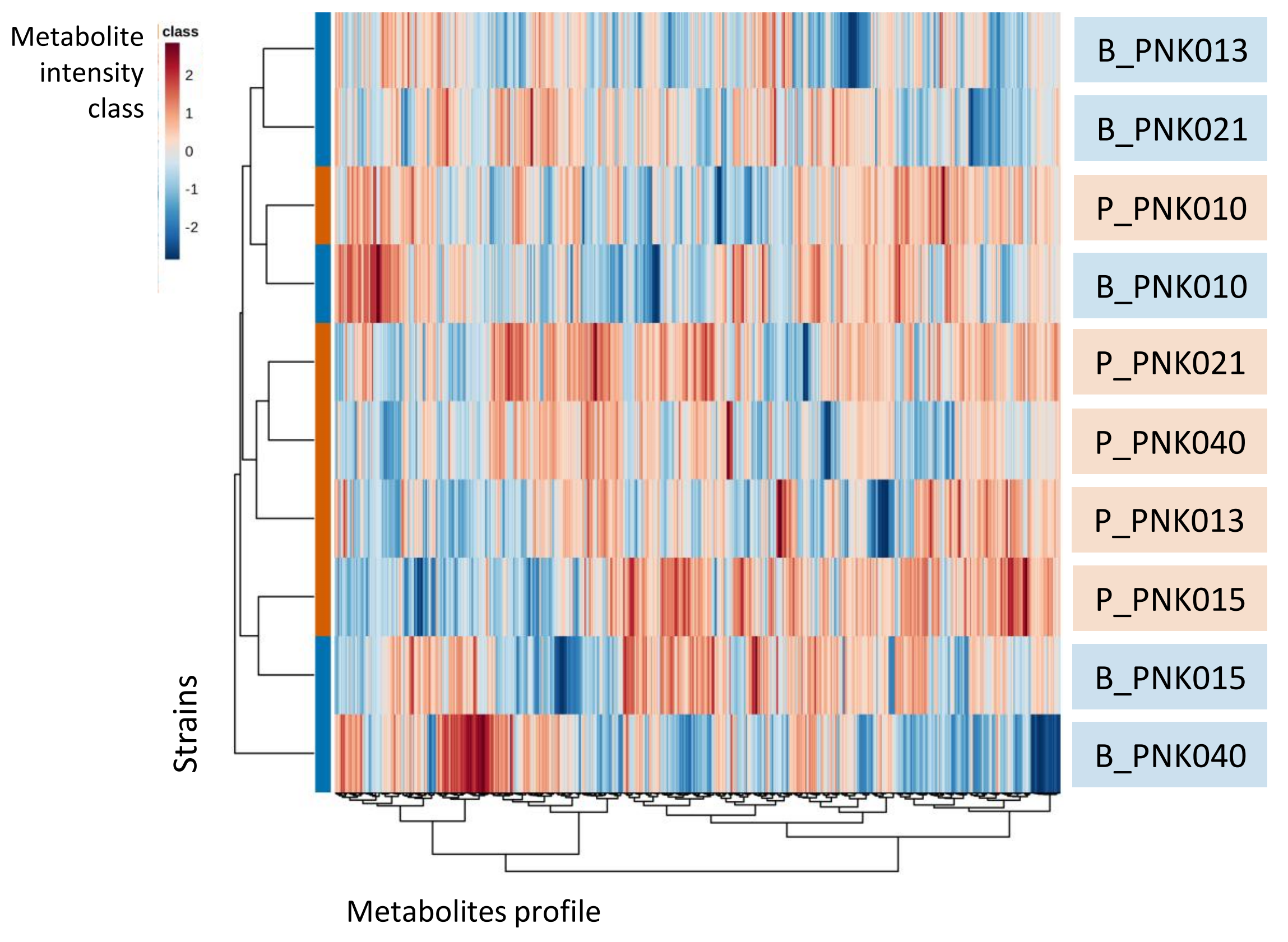

Figure S2. Heatmap of metabolomic profiles for algal strains growing for a year in (**a**) laboratory conditions and (**b**) in the environment in relation to the substrate type: plaster (P) vs. brick (B); hierarchical clustering was based on normalized metabolite intensities, the color intensity represents metabolite abundance (scaled values), and the strains refer to the algal taxa: (PNK010*) Chloroidium saccharophilum*, (PNK013) *Klebsormidium nitens*, (PNK015) *Bracteacoccus minor*, (PNK021) *Diplosphaera chodatii*, (PNK040) *Stichocccus bacillaris*.
