# Supplementary material for: Assessing the potential for biogeochemical deterioration of building materials by green algae in temperate climate via an integrated metabolomic approach
Source: Sci Rep. 2026 Jun 19;16:28186. doi: 10.1038/s41598-026-58452-8 (PMC13554127; doi:10.1038/s41598-026-58452-8)
Supplement: Supplementary file 2 [file 41598_2026_58452_MOESM2_ESM.pdf]

Chloroidium saccharophilum PNK010

Plaster

Laboratory

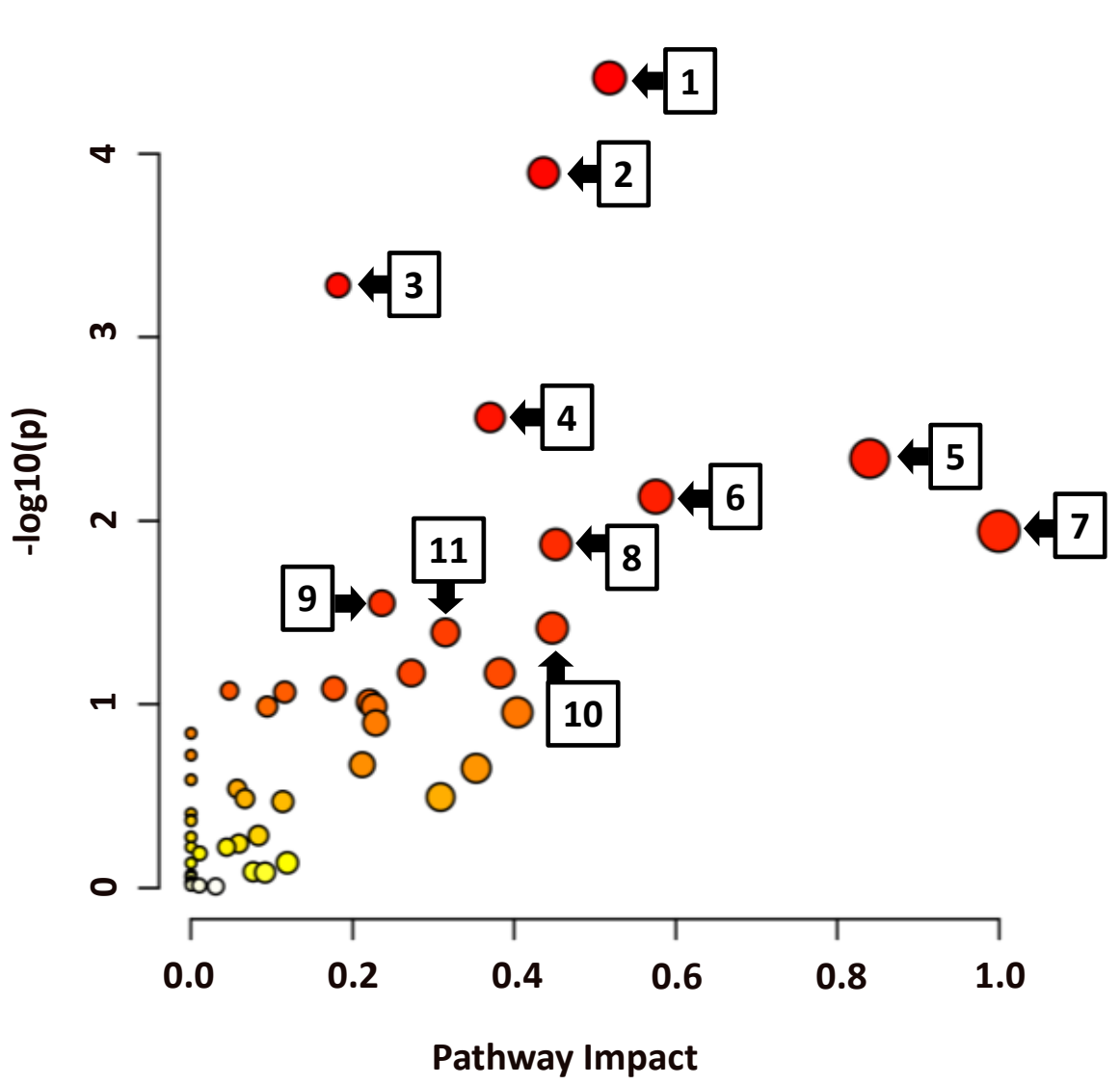

Environment

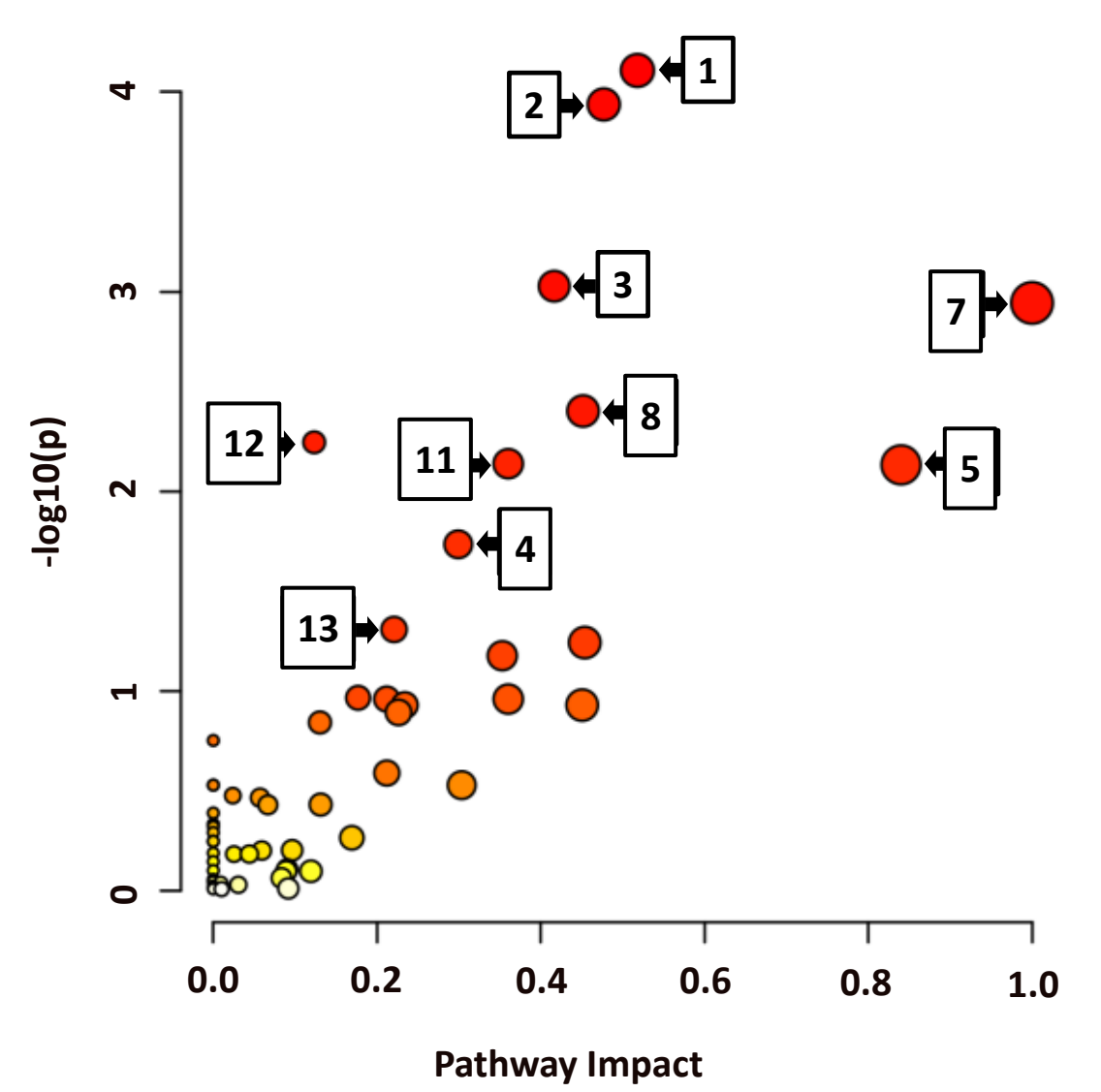

Brick

Laboratory

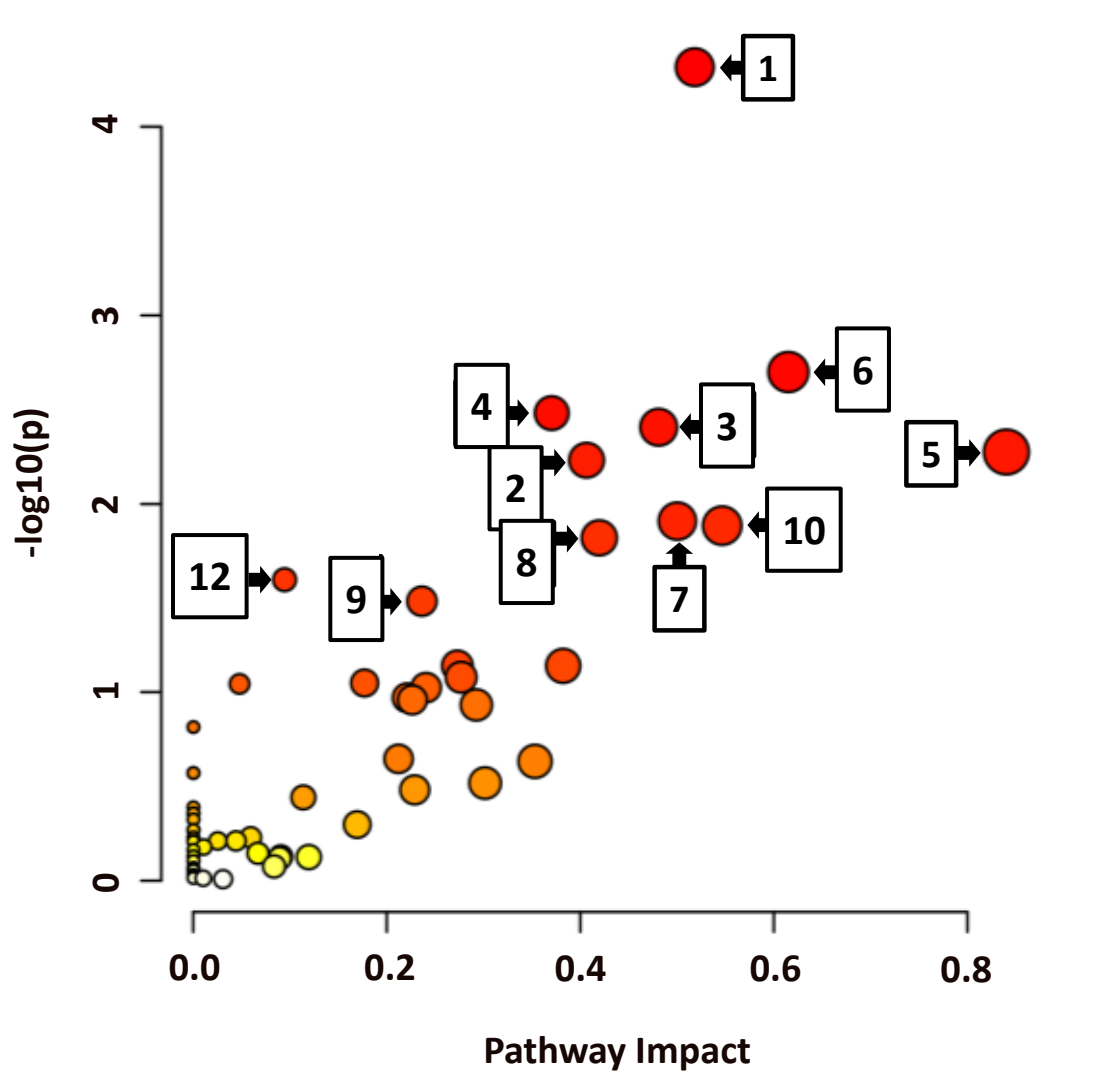

Environment

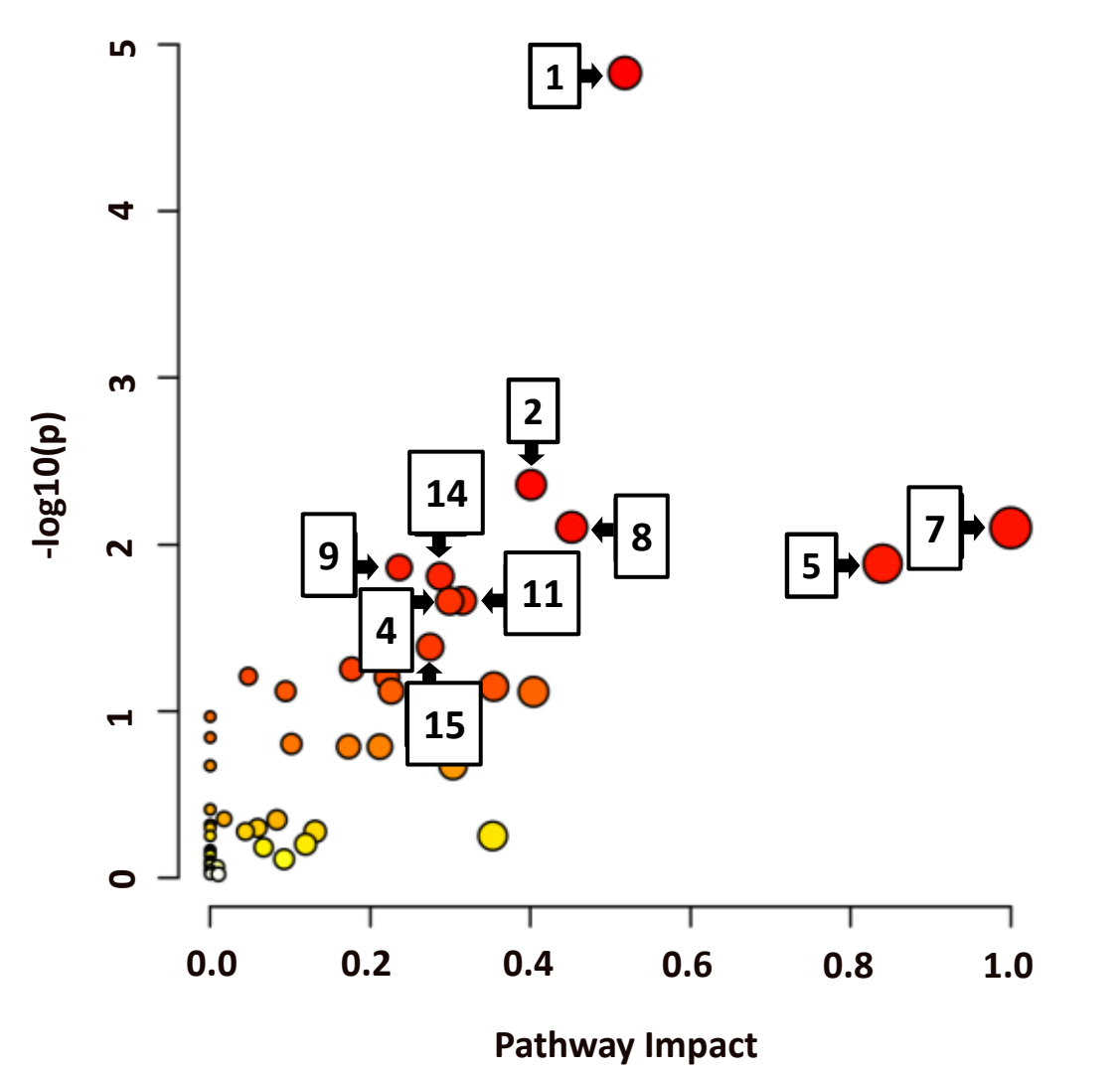

Figure S3. Topology of metabolic pathways detected in *Chloroidium saccharophilum* PNK010 strain growing for a year on plaster and brick under laboratory and environmental conditions. The plots show the most significant pathways (–log10(p) > 1.3; p-value ≤ 0.05) based on metabolites with a fold change > 2 relative to the corresponding control samples. Numbered markers correspond to the following pathways: **[1]** Arginine biosynthesis; **[2]** Purine metabolism; **[3]** One carbon pool by folate; **[4]** Glyoxylate and dicarboxylate metabolism; **[5]** Alanine, aspartate, and glutamate metabolism; **[6]** Glutathione metabolism; **[7]** Isoquinoline alkaloid biosynthesis; **[8]** Tyrosine metabolism; **[9]** Cysteine and methionine metabolism; **[10]** Glycine, serine, and threonine metabolism; **[11]** Glycerophospholipid metabolism; **[12]** Nicotinate and nicotinamide metabolism; **[13]** Arginine and proline metabolism; **[14]** Galactose metabolism; **[15]** Pyrimidine metabolism.

Klebsormidium nitens PNK013

Plaster

Laboratory

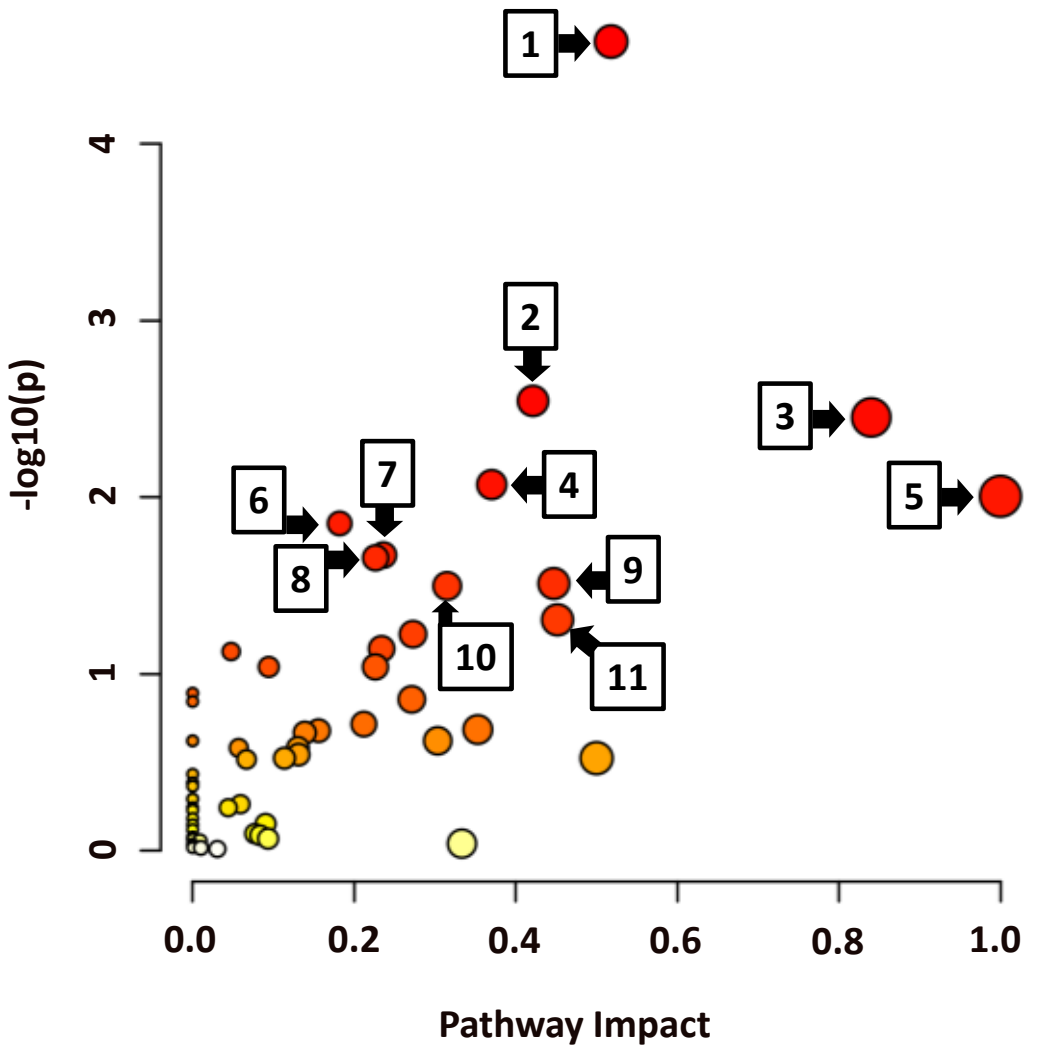

Environment

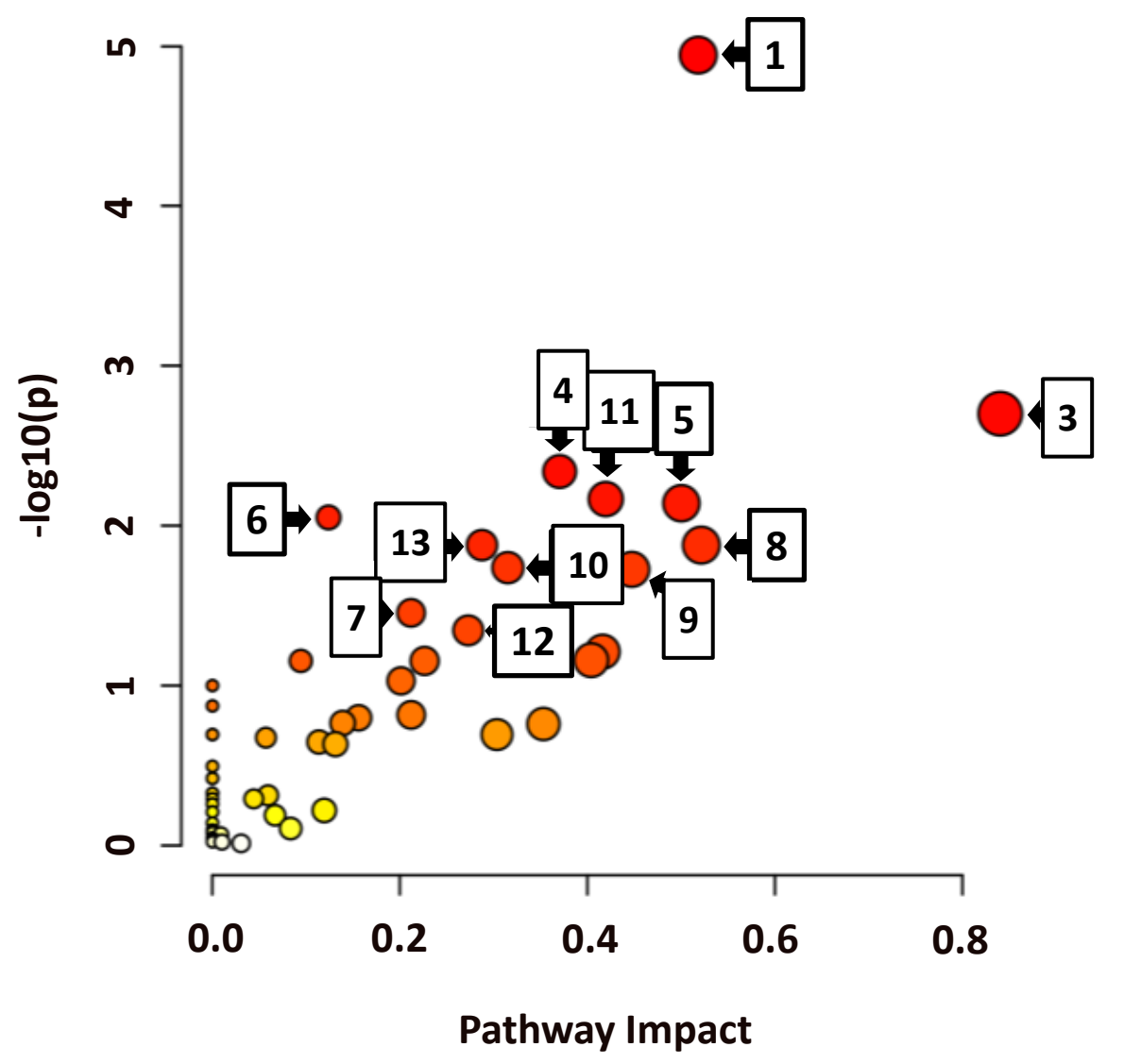

Brick

Laboratory

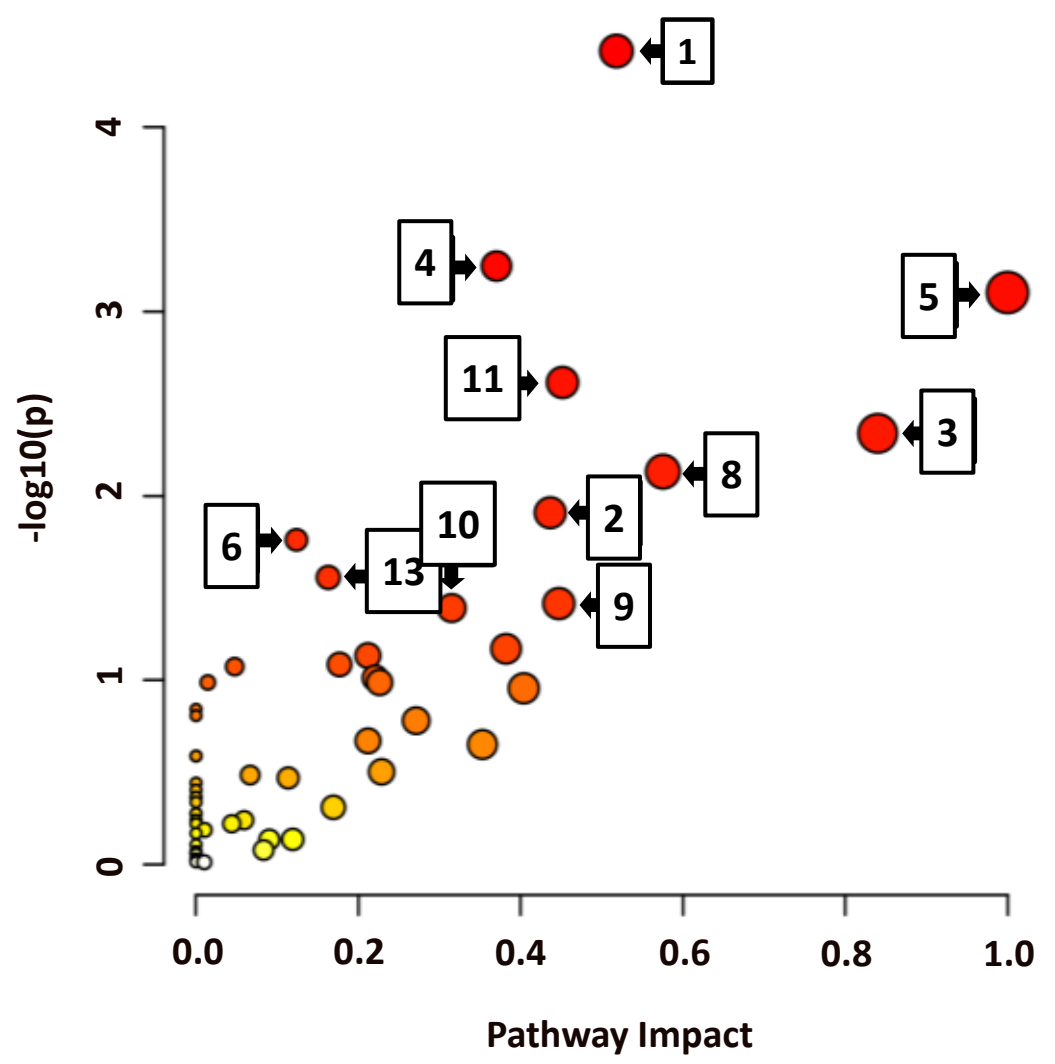

Environment

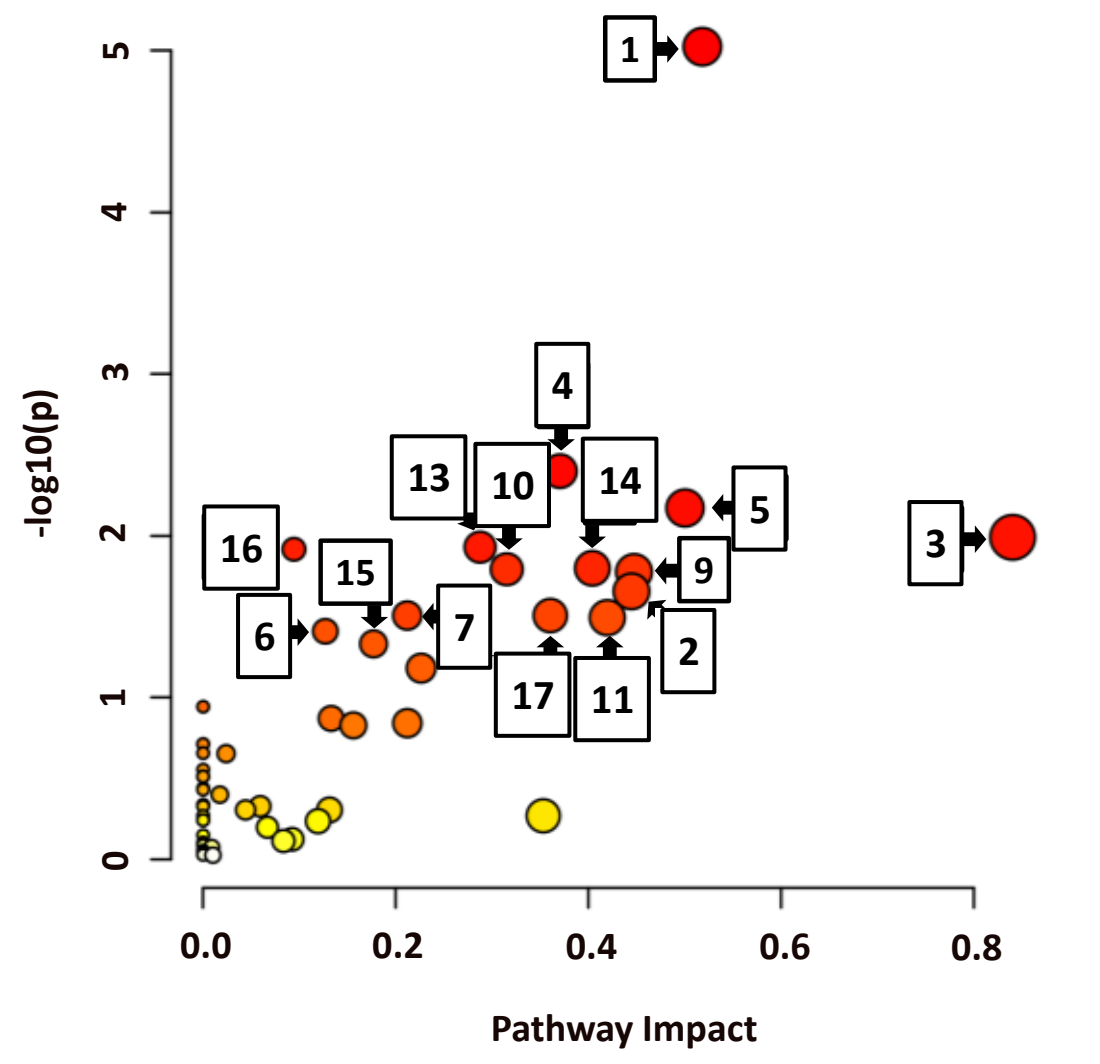

Figure S4. Topology of metabolic pathways detected in *Klebsormidium nitens* PNK013 strain growing for a year on plaster and brick under laboratory and environmental conditions. The plots show the most significant pathways (–log10(p) > 1.3; p-value ≤ 0.05) based on metabolites with a fold change > 2 relative to the corresponding control samples. Numbered markers correspond to the following pathways: **[1]** Arginine biosynthesis; **[2]** Purine metabolism; **[3]** Alanine, aspartate, and glutamate metabolism; **[4]** Glyoxylate and dicarboxylate metabolism; **[5]** Isoquinoline alkaloid biosynthesis; **[6]** One carbon pool by folate; **[7]** Cysteine and methionine metabolism; **[8]** Glutathione metabolism; **[9]** Glycine, serine and threonine metabolism; **[10]** Glycerophospholipid metabolism; **[11]** Tyrosine metabolism; **[12]** Nitrogen metabolism; **[13]** Galactose metabolism; **[14]** Starch and sucrose metabolism; **[15]** Citrate cycle (TCA cycle); **[16]** Nicotinate and nicotinamide metabolism; **[17]** Pyrimidine metabolism.

Bracteacoccus minor PNK015

Plaster

Laboratory **Environment**

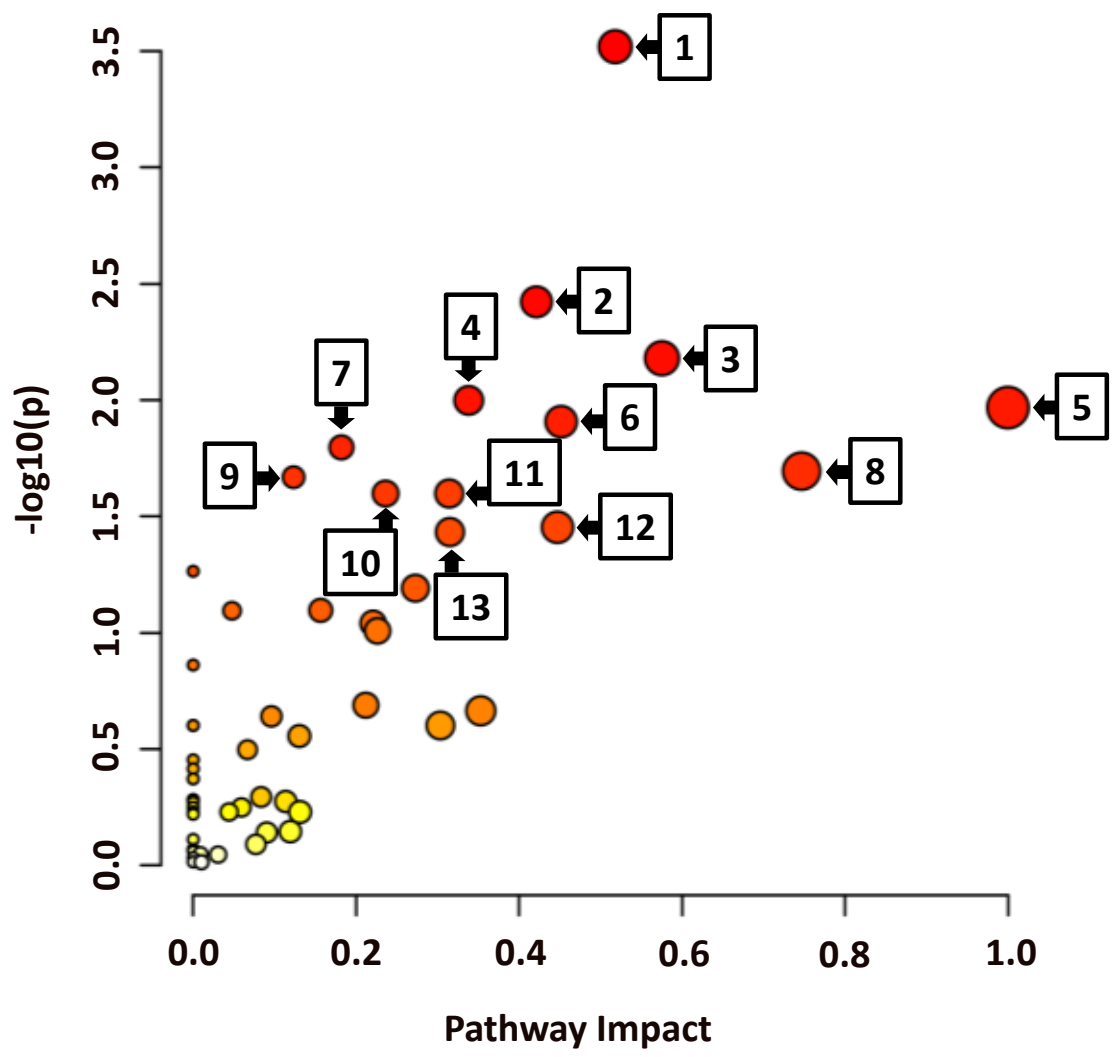

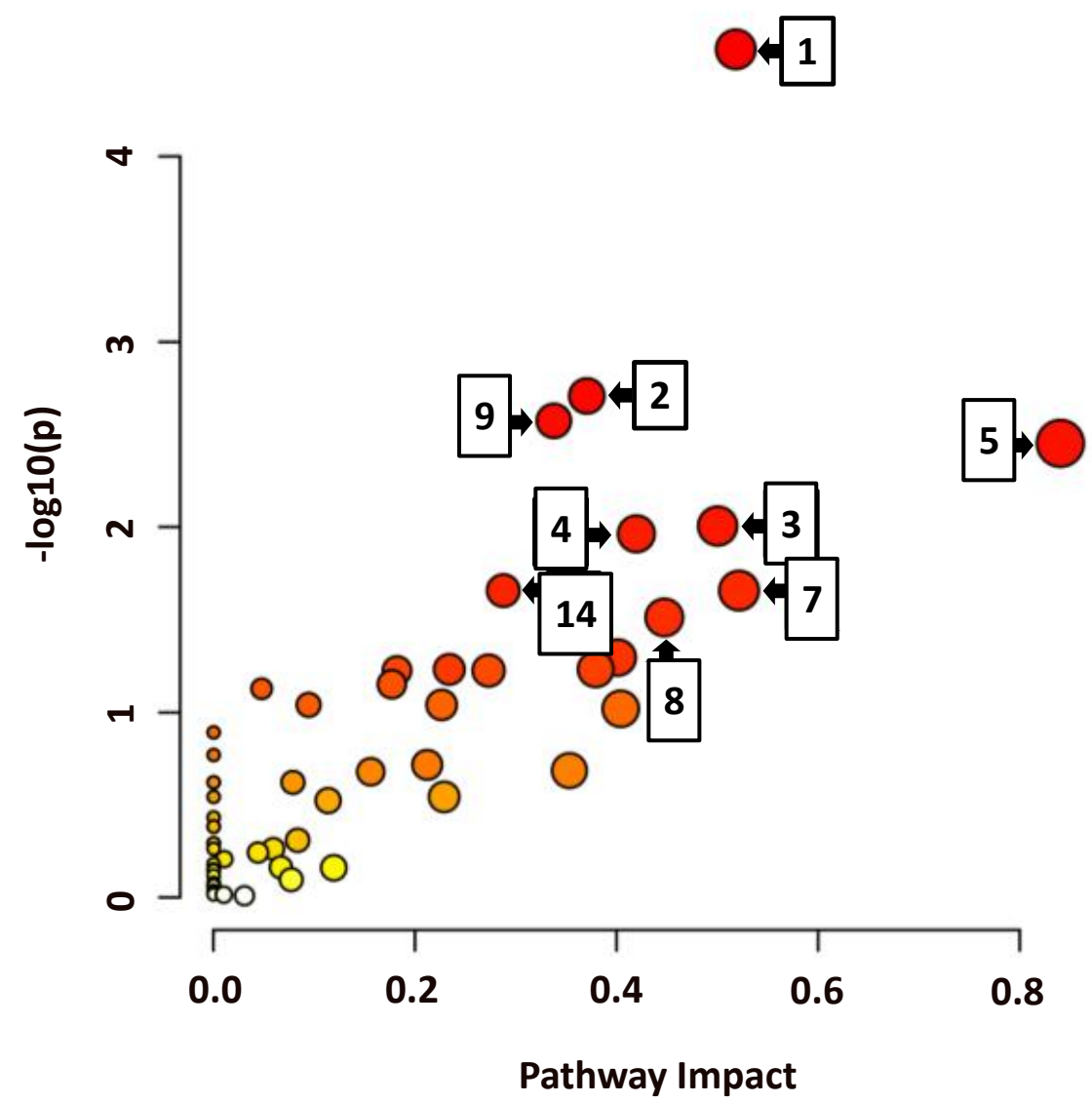

Brick

Laboratory **Environment**

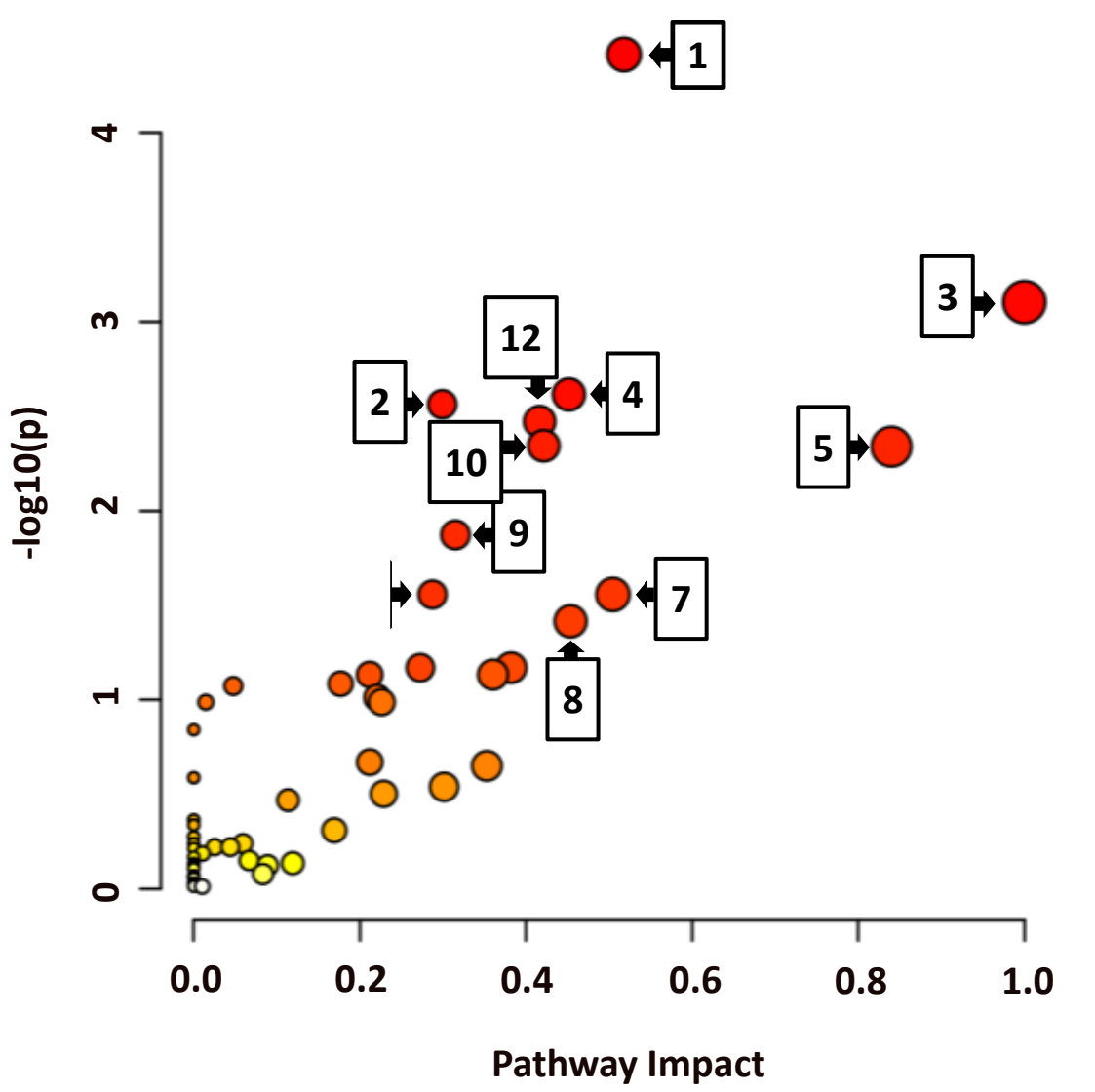

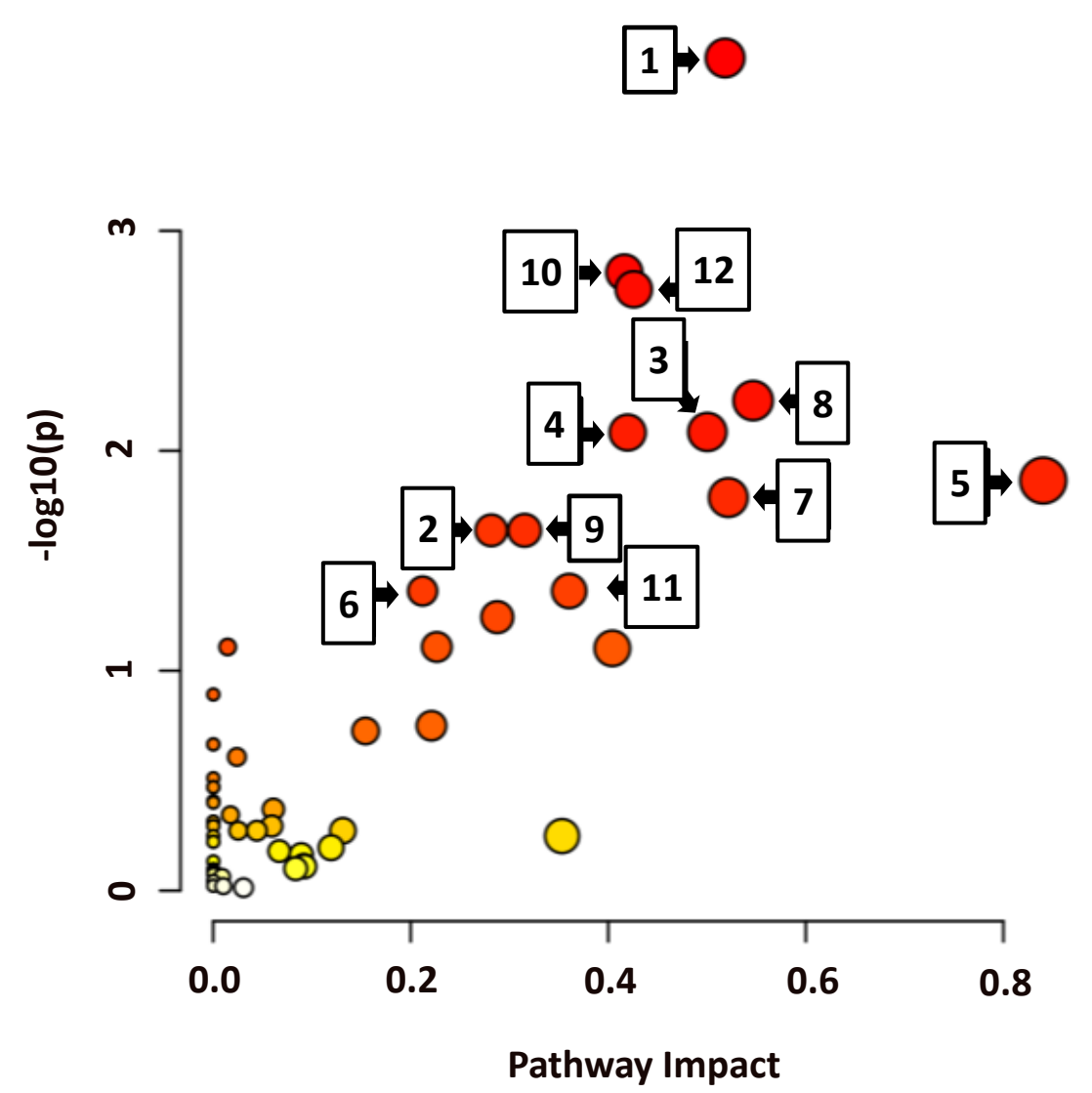

Figure S5. Topology of metabolic pathways detected in *Bracteacoccus minor* PNK015 strain growing for a year on plaster and brick under laboratory and environmental conditions. The plots show the most significant pathways (–log10(p) > 1.3; p-value ≤ 0.05) based on metabolites with a fold change > 2 relative to the corresponding control samples. Numbered markers correspond to the following pathways: **[1]** Arginine biosynthesis; **[2]** Glyoxylate and dicarboxylate metabolism; **[3]** Isoquinoline alkaloid biosynthesis; **[4]** Tyrosine metabolism; **[5]** Alanine, aspartate, and glutamate metabolism; **[6]** Cysteine and methionine metabolism; **[7]** Glutathione metabolism; **[8]** Glycine, serine, and threonine metabolism; **[9]** Glycerophospholipid metabolism; **[10]** Purine metabolism; **[11]** Pyrimidine metabolism; **[12]** One carbon pool by folate; **[13]** Nitrogen metabolism; **[14]** Galactose metabolism.

Diplosphaera chodatii PNK021

Plaster

Laboratory

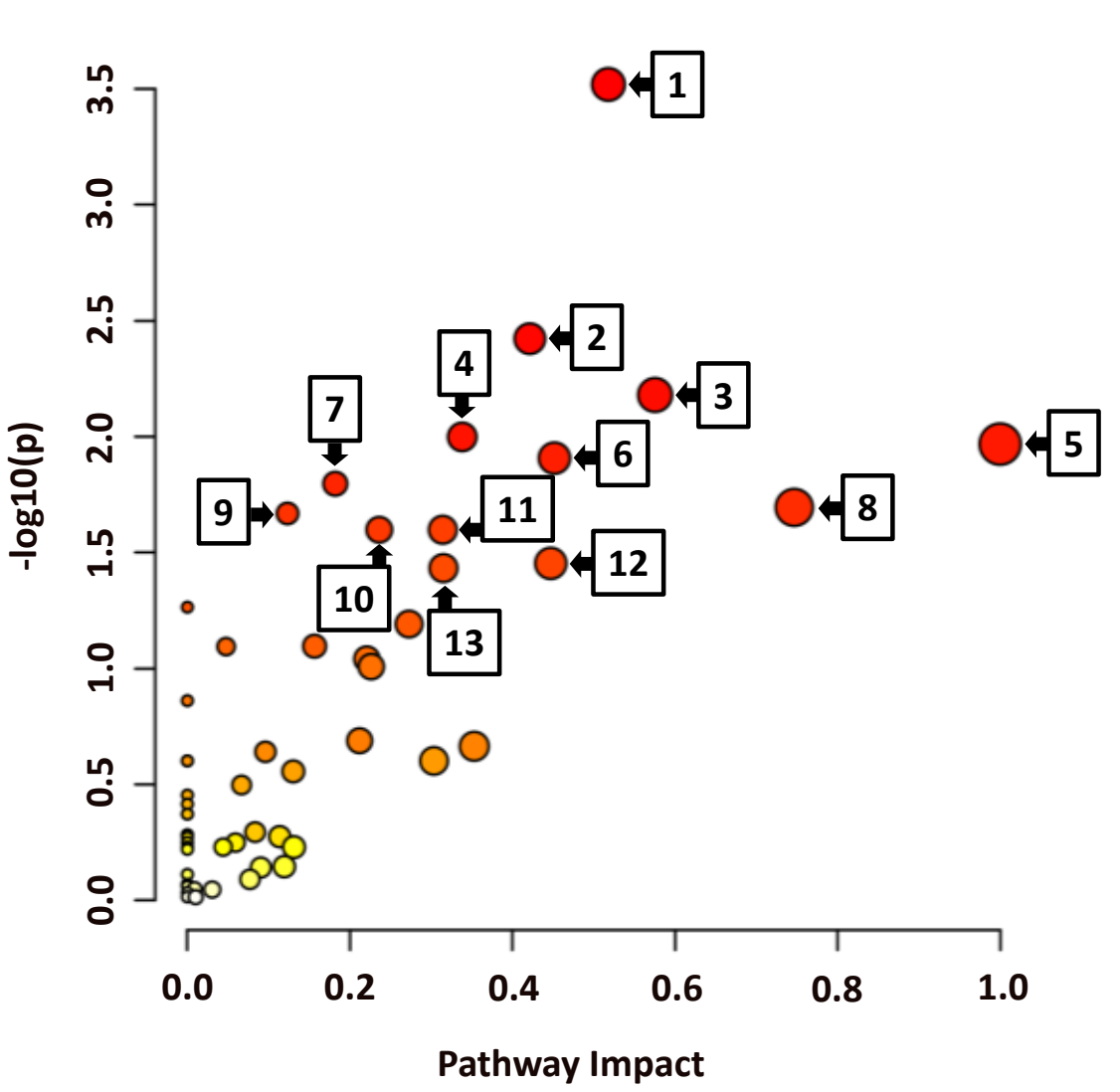

Environment

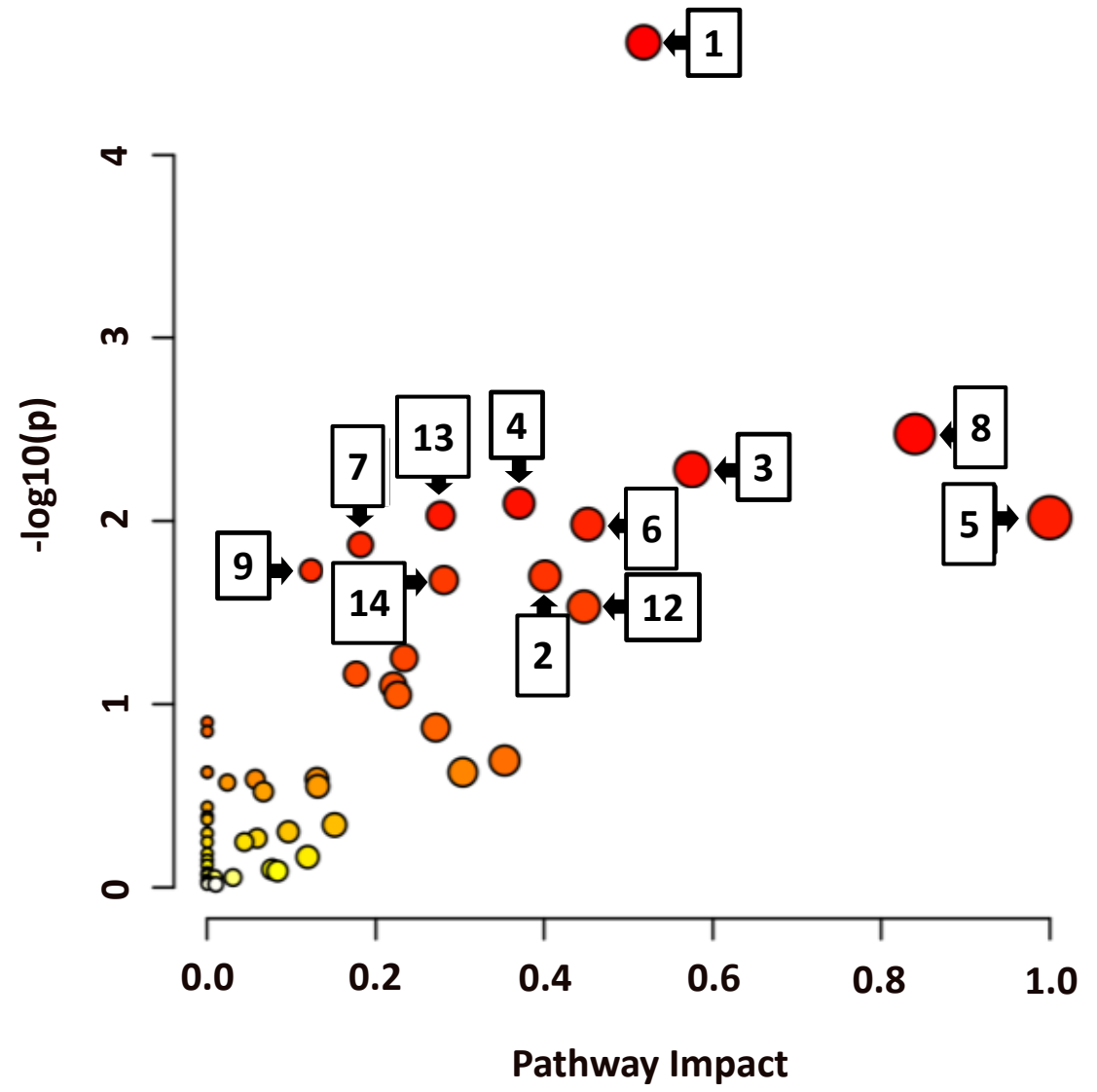

Brick

Laboratory

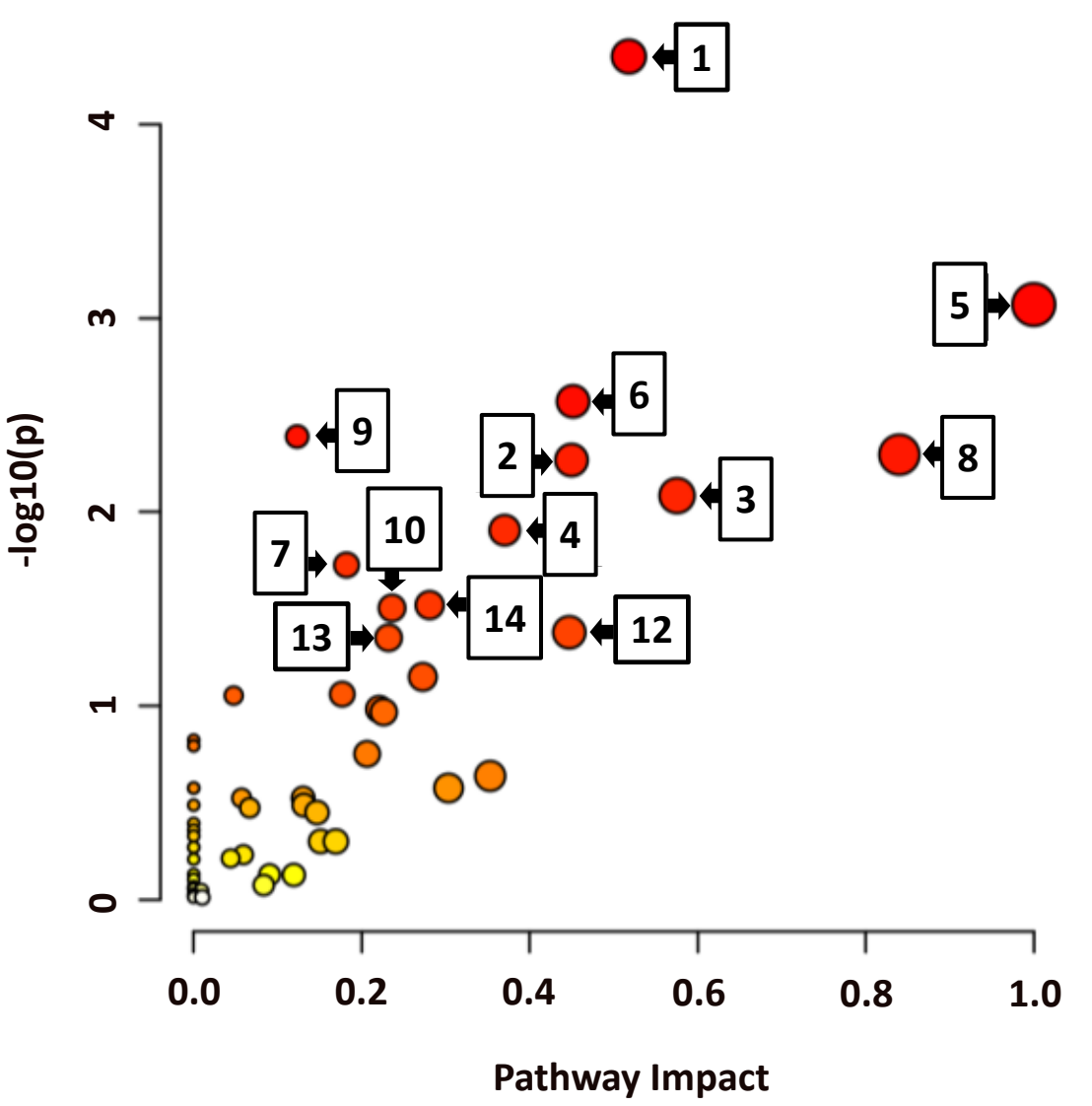

Environment

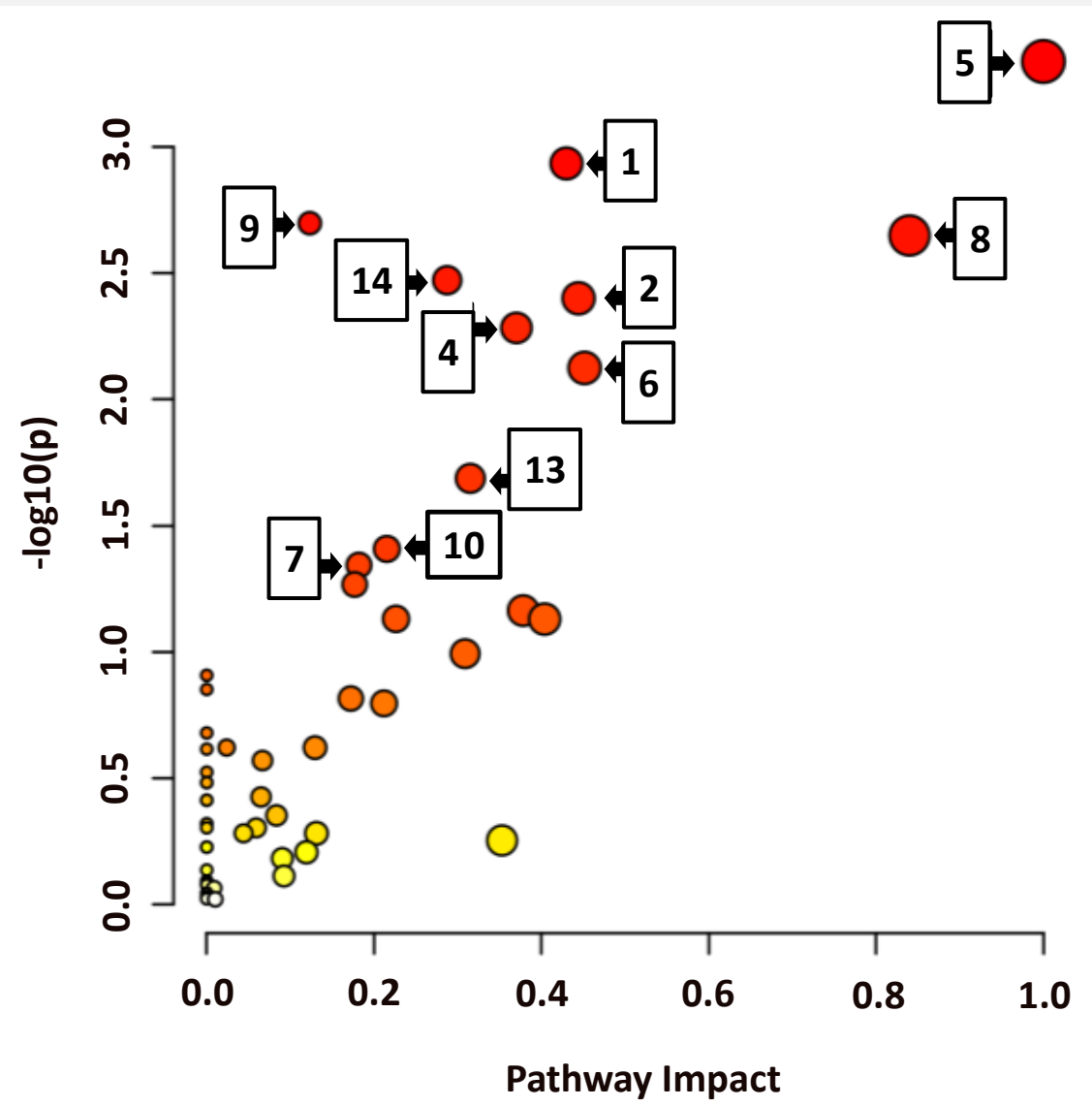

Figure S6. Topology of metabolic pathways detected in *Diplosphaera chodatii* PNK021 strain growing for a year on plaster and brick under laboratory and environmental conditions. The plots show the most significant pathways (–log10(p) > 1.3; p-value ≤ 0.05) based on metabolites with a fold change > 2 relative to the corresponding control samples. Numbered markers correspond to the following pathways: **[1]** Arginine biosynthesis; **[2]** Purine metabolism; **[3]** Glutathione metabolism; **[4]** Glyoxylate and dicarboxylate metabolism; **[5]** Isoquinoline alkaloid biosynthesis; **[6]** Tyrosine metabolism; **[7]** One carbon pool by folate; **[8]** Alanine, aspartate, and glutamate metabolism; **[9]** Nicotinate and nicotinamide metabolism; **[10]** Cysteine and methionine metabolism; **[11]** Pyrimidine metabolism; **[12]** Glycine, serine, and threonine metabolism; **[13]** Glycerophospholipid metabolism; **[14]** Galactose metabolism.

Stichococcus bacillaris PNK040

Plaster

Laboratory

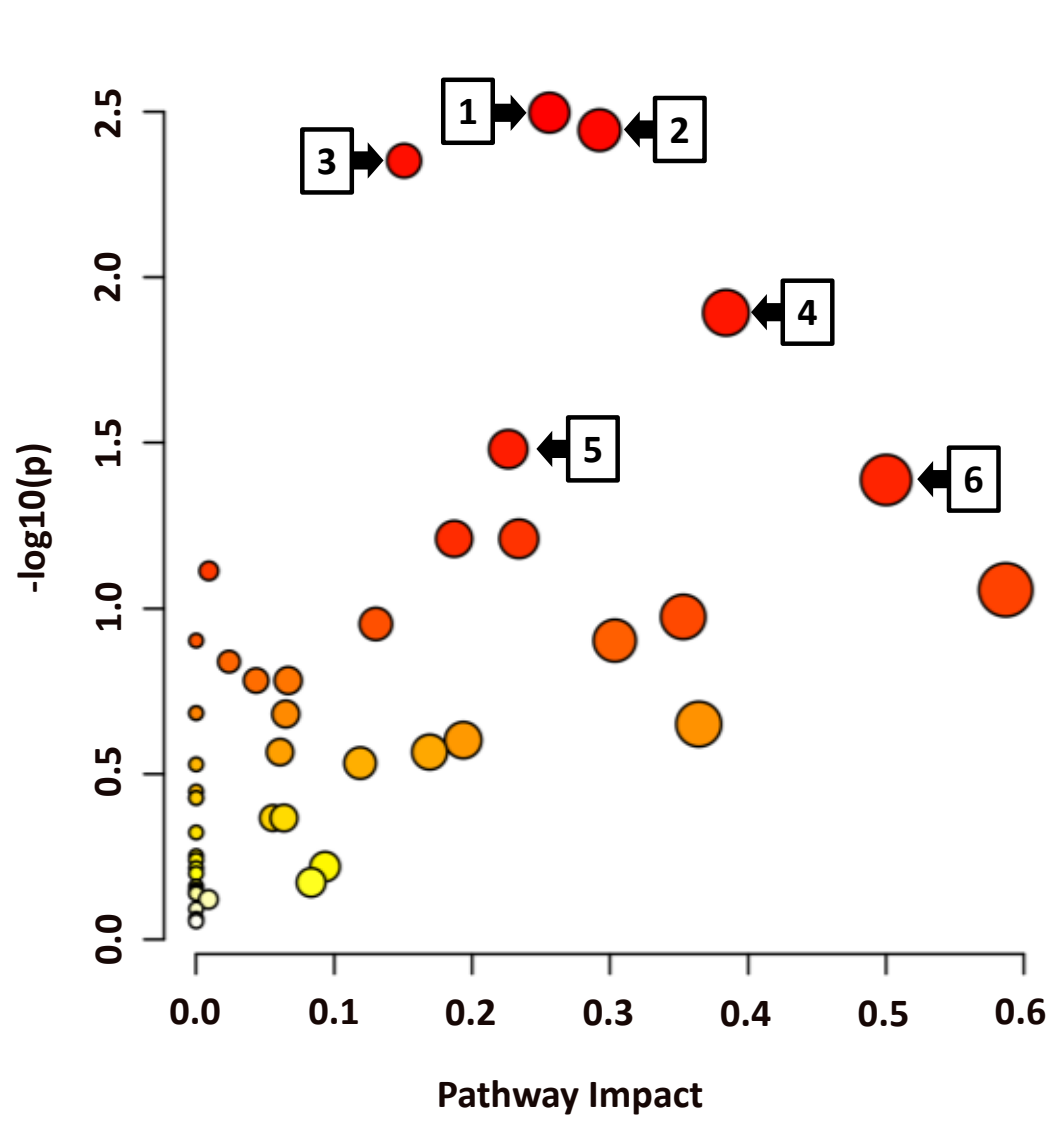

Environment

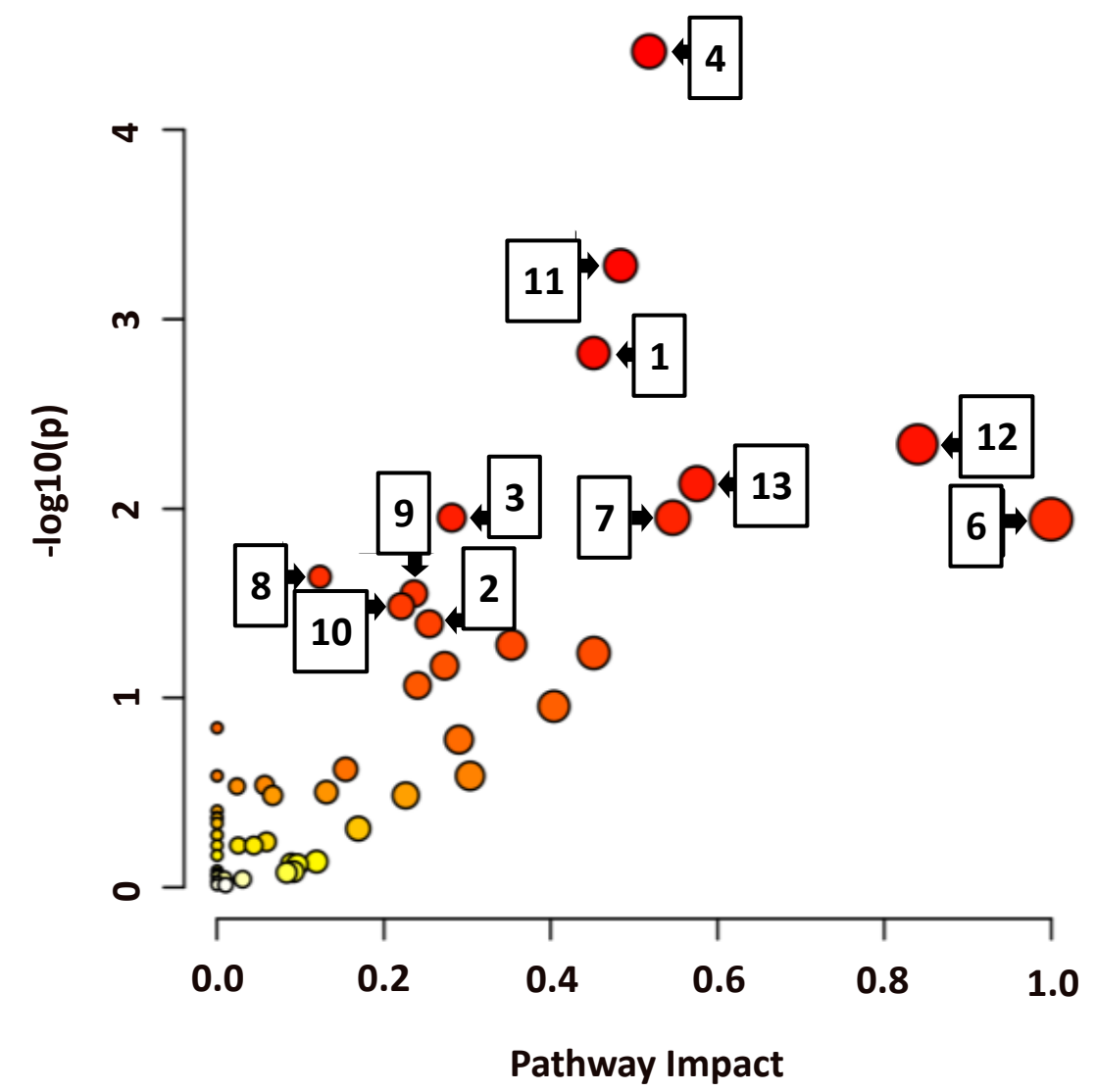

Brick

Laboratory

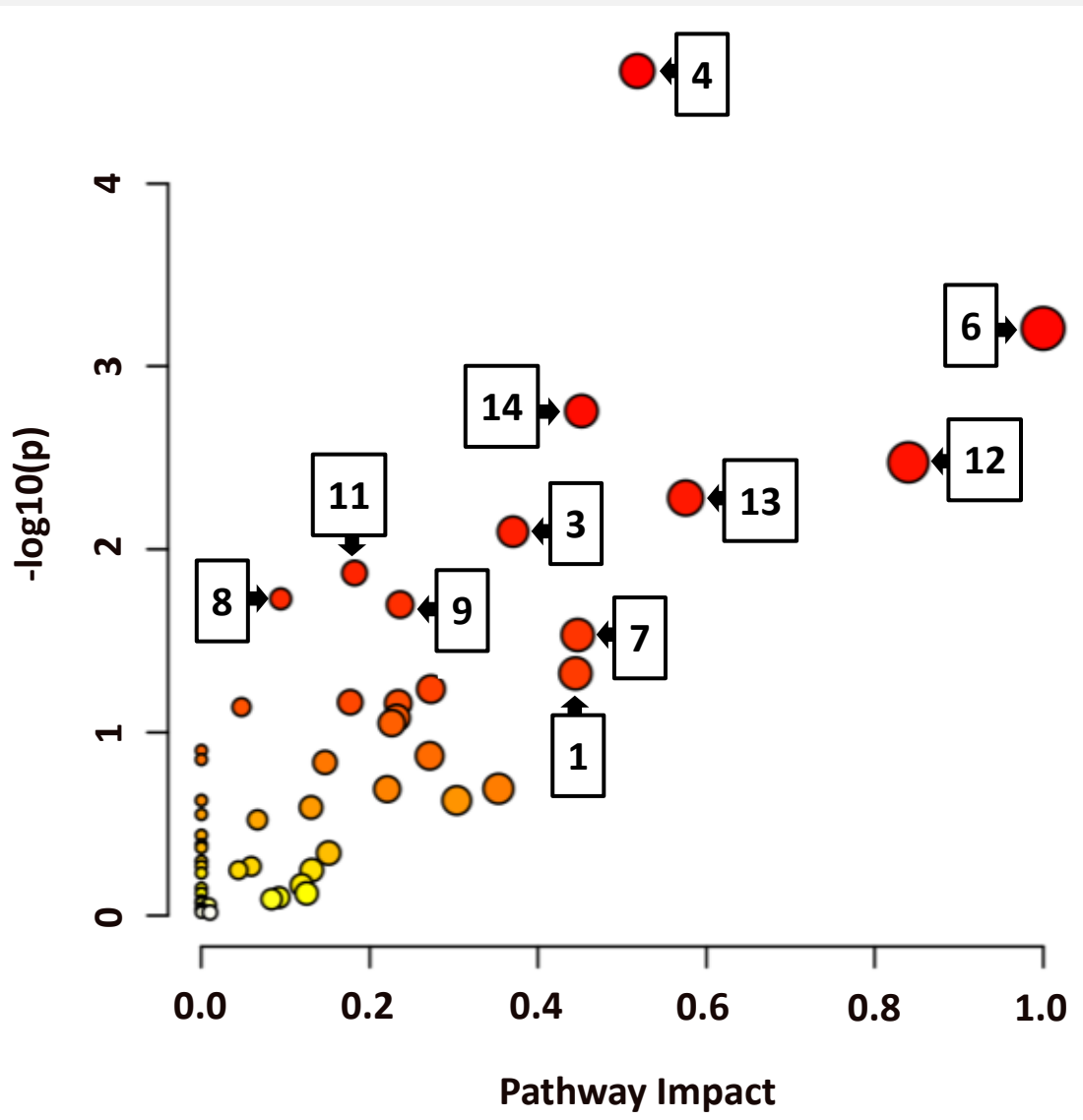

Environment

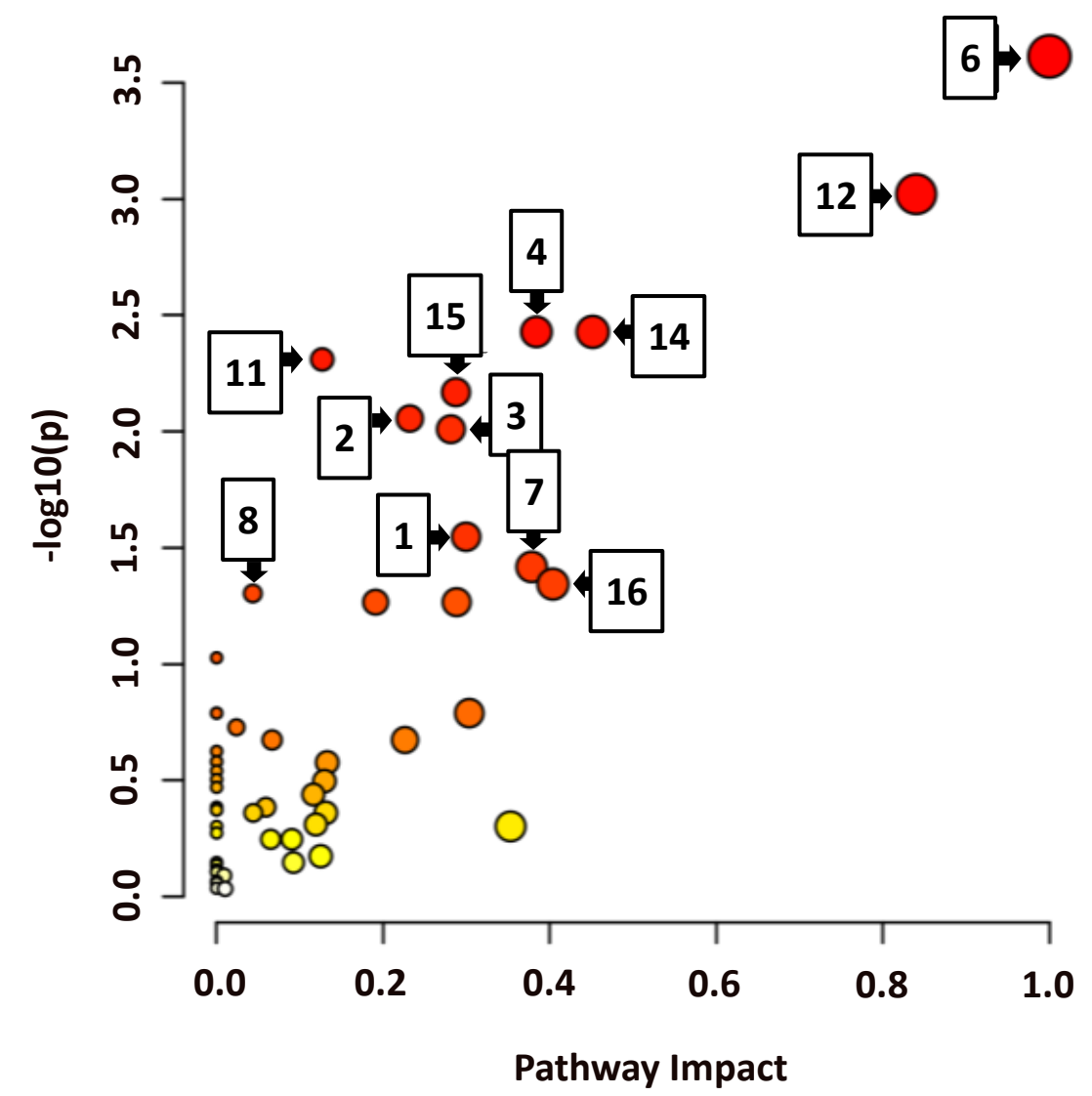

Figure S7. Topology of metabolic pathways detected in *Stichococcus bacillaris* PNK040 strain growing for a year on plaster and brick under laboratory and environmental conditions. The plots show the most significant pathways (–log10(p) > 1.3; p-value ≤ 0.05) based on metabolites with a fold change > 2 relative to the corresponding control samples. Numbered markers correspond to the following pathways: **[1]** Purine metabolism; **[2]** Glycerophospholipid metabolism; **[3]** Glyoxylate and dicarboxylate metabolism; **[4]** Arginine biosynthesis; **[5]** Riboflavin metabolism; **[6]** Isoquinoline alkaloid biosynthesis; **[7]** Glycine, serine, and threonine metabolism; **[8]** Nicotinate and nicotinamide metabolism; **[9]** Cysteine and methionine metabolism; **[10]** Arginine and proline metabolism; **[11]** One carbon pool by folate; **[12]** Alanine, aspartate, and glutamate metabolism; **[13]** Glutathione metabolism; **[14]** Tyrosine metabolism; **[15]** Galactose metabolism; **[16]** Starch and sucrose metabolism.
